# Targeted cystatin C testing for large negative eGFR discordance in adults with diabetes: development and external validation of a machine-learning model

**DOI:** 10.3389/fendo.2026.2002540

**Published:** 2026-10-29

**Authors:** Xuanxuan Wang, Mengyu Zhang, Ke Zhang, Yuanhong Xu, Yingying Chen, Yuanyuan Hu

**Affiliations:** 1 Department of Clinical Laboratory, The First Affiliated Hospital of Anhui Medical University, Hefei, Anhui, China; 2 Department of Clinical Laboratory, Wanbei Coal Electric Group General Hospital, Suzhou, Anhui, China; 3 Department of Hematology, The First Affiliated Hospital of Anhui Medical University, Hefei, Anhui, China

**Keywords:** cystatin C, diabetes, eGFR discordance, estimated glomerular filtration rate, external validation, machine learning, targeted testing

## Abstract

**Background:**

Large negative discordance between cystatin C- and creatinine-based estimated glomerular filtration rate (eGFR) may reveal clinically relevant renal-function differences that are not apparent from creatinine alone. When universal cystatin C testing is impractical, a method to prioritize patients for additional testing may improve testing efficiency.

**Methods:**

We conducted a retrospective two-center study of adults with diabetes. Large negative eGFR discordance (D2) was defined as eGFRcys/eGFRcr <0.70. Seven machine-learning algorithms were evaluated in a development cohort of 1,170 participants (214 D2-positive), followed by SHAP-guided feature reduction. The final model was applied unchanged to an independent external validation cohort of 1,619 participants (112 D2-positive). Performance was compared with a prespecified clinical core (age, sex, and eGFRcr); additional analyses evaluated logistic regression using eGFRcr alone or with blood urea nitrogen and albumin. Prespecified cystatin C testing allocations of 10%-50% were evaluated using model-based risk ranking.

**Results:**

The final nine-predictor XGBoost model achieved an AUROC of 0.863 (95% CI, 0.833-0.889) and an AUPRC of 0.600 (95% CI, 0.532-0.668) in development. In independent external validation, AUROC was 0.806 (95% CI, 0.769-0.840) and AUPRC was 0.258 (95% CI, 0.193-0.337), compared with 0.661 and 0.126, respectively, for the clinical core. At the 20% testing allocation, risk-guided testing identified 61.2% of D2 cases in development and 55.4% externally, with 3.06-fold and 2.77-fold enrichment over random testing. At this allocation in external validation, XGBoost identified 62 D2 cases versus 43 with the three-variable model (recall difference, 17.0 percentage points; 95% CI, 8.6-27.2). However, raw external probabilities overestimated D2 risk (mean predicted probability, 29.9% vs. observed prevalence, 6.9%), and the Brier score was higher than that of the clinical core (0.144 vs. 0.130).

**Conclusions:**

In two cohorts of adults with diabetes who underwent paired testing, routine clinical laboratory data supported prioritization for large negative eGFR discordance. Model-based ranking may help target cystatin C testing in similar settings, but raw probabilities require local calibration before threshold-based decisions and prospective evaluation is needed before clinical implementation.

## Introduction

Assessment of glomerular filtration rate (GFR) and albuminuria is central to the evaluation of chronic kidney disease (CKD) in adults with diabetes (1, 2). Creatinine-based estimated GFR (eGFRcr) is routinely available, but non-GFR determinants of creatinine can affect its interpretation. Cystatin C provides complementary information, although it is also influenced by non-GFR factors (2, 3). KDIGO guidance favors combined creatinine-cystatin C estimates for GFR categorization when cystatin C is available, particularly when eGFRcr is less accurate and GFR informs clinical decisions (2). A lower cystatin C-based eGFR (eGFRcys), however, does not establish which estimate is closer to measured GFR; when eGFRcr and eGFRcys are discordant, the combined creatinine-cystatin C equation is generally more accurate than either single-marker estimate (4).

Discordance between eGFRcr and eGFRcys has prognostic relevance. Recent individual-level and aggregate meta-analyses have associated large negative discordance with higher risks of mortality, cardiovascular events, heart failure, and kidney failure (5, 6), and a large routine-care cohort reported similar associations across multiple adverse outcomes (7). Cohort studies in adults with diabetes have further linked differences between the two estimates with microvascular complications and incident albuminuria (8, 9). These findings provide a clinical rationale for identifying patients likely to have large negative eGFR discordance.

When cystatin C testing capacity is constrained, a practical question is whom to test first. Recent clinical guidance and review literature recognize that assay availability and implementation barriers can limit broader use of cystatin C (10). Peralta and colleagues developed and externally validated a score for occult-reduced eGFR and evaluated the proportion of cases detected at different testing allocations (11), while a UK Biobank study modeled the prevalence and predictors of differences between creatinine- and cystatin C-based eGFR (12). More broadly, recent diabetes-focused machine-learning studies have shown that routine clinical and laboratory variables can support identification or risk prediction of diabetic kidney disease and related renal phenotypes (13–16). These studies establish a basis for prediction-guided testing, but they target different kidney phenotypes and populations. A diabetes-focused assessment of substantial relative eGFR discordance can therefore clarify whether a compact routine-laboratory model retains performance across hospital settings and concentrates affected patients within limited testing allocations.

We therefore developed and independently externally validated a machine-learning model for large negative eGFR discordance in adults with diabetes, defined *a priori* as eGFRcys/eGFRcr <0.70. The intended application was prioritization of cystatin C measurement using demographic and laboratory information available in routine care. We compared the final model with a clinical core comprising age, sex, and eGFRcr, characterized model behavior, and quantified case capture, testing yield, and enrichment across prespecified testing allocations. We also examined related renal phenotypes among model-selected participants who did not meet the primary discordance definition.

## Methods

### Study population and data collection

This retrospective two-center study included adults aged ≥18 years with a recorded clinical diagnosis of diabetes and paired serum creatinine and cystatin C measurements. The development cohort was assembled at Wanbei Hospital from May 1, 2022, through November 30, 2023, and the independent external validation cohort was drawn from available 2024 clinical records at the First Affiliated Hospital of Anhui Medical University. Diabetes type could not be reliably classified across both centers. Reporting was informed by TRIPOD+AI guidance (17).

Each participant contributed the earliest eligible paired measurement within the study period. Serum creatinine and cystatin C were measured from the same blood specimen using Atellica CH 930 analyzers (Siemens Healthineers) at both hospitals. Serum creatinine was measured using the sarcosine oxidase enzymatic method with reagents manufactured by Anhui Tongzhi Biological Engineering Co., Ltd., with calibration traceable to GBW(E)091004 and the isotope dilution mass spectrometry (IDMS) reference system. Serum cystatin C was measured using a latex-enhanced immunoturbidimetric assay with reagents manufactured by Anhui Iprocom Biotechnology Co., Ltd., with calibration traceable to ERM-DA471/IFCC. Both hospitals used the same analytical instrument model, reagent products, and measurement methods, without changes during their respective study periods. Chemistry and lipid measurements were obtained from the index specimen, whereas complete blood count and glycated hemoglobin measurements were obtained from the nearest eligible panel from 7 days before through the index date. Modeling eligibility additionally required valid measured white blood cell count, hemoglobin, and platelet count; missing values were not imputed to satisfy this eligibility rule. Detailed cohort-construction, linkage, and quality-control procedures are provided in Supplementary Methods 1.

### Definition of eGFR discordance

Creatinine-based estimated glomerular filtration rate (eGFRcr) was calculated using the 2021 CKD-EPI creatinine equation, and cystatin C-based eGFR (eGFRcys) using the 2012 CKD-EPI cystatin C equation (18, 19). Serum creatinine values reported in micromol/L were converted to mg/dL by division by 88.4, and cystatin C was expressed in mg/L. The primary phenotype (D2) was defined as eGFRcys/eGFRcr <0.70, corresponding to eGFRcys being more than 30% lower than eGFRcr. This relative-discordance threshold is consistent with previous outcome research (5) but was not intended as a CKD diagnosis or a clinical action cutoff.

Two related phenotypes were used for secondary characterization. D1 was defined as eGFRcys − eGFRcr ≤−15 mL/min/1.73 m². D3 was defined as eGFRcys <60 mL/min/1.73 m² among participants with eGFRcr ≥60 mL/min/1.73 m²; only this eligible subset contributed to D3 denominators. All three definitions were based on a single paired measurement and were therefore treated as renal-function phenotypes rather than chronicity-confirmed CKD diagnoses or independently adjudicated kidney disease.

### Candidate variables and data preprocessing

An initial set of 49 demographic and routinely available laboratory variables was considered. After review of cross-center predictor availability, missingness, measurement comparability, and clinical redundancy, 28 variables were retained as the fixed Full feature pool for model development. The external cohort contributed predictor-availability and measurement-harmonization information for this feasibility review, whereas model fitting, and SHAP-based ranking used development-cohort data. Variables with >25% missingness in either modeling cohort were excluded. Cystatin C, eGFRcys, and other cystatin C-derived phenotype measurements were not eligible predictors. Available measurements and missing-data percentages for all 49 candidate variables in each cohort are reported in **Supplementary Table S1**, with the pre-redundancy correlation structure shown in **Supplementary Figure S1**.

Missing predictor values were imputed using medians estimated within each training fold. Logistic regression, support vector machine, and k-nearest neighbors additionally underwent training-fold standardization. No class weighting, oversampling, or synthetic resampling was used.

### Model development and comparison

Seven classification algorithms were evaluated using the identical 28-variable Full feature pool: logistic regression, support vector machine, k-nearest neighbors, decision tree, random forest, XGBoost, and LightGBM. Model development used stratified five-fold cross-validation with shuffling and a fixed random seed of 42. Hyperparameter search spaces and selected values are reported in **Supplementary Table S3**.

Average precision, reported as the area under the precision–recall curve (AUPRC), was the prespecified primary metric for model selection, with the area under the receiver operating characteristic curve (AUROC) and Brier score as secondary measures. Performance was summarized from pooled patient-level out-of-fold predictions. Because hyperparameter selection and out-of-fold performance estimation used the same five-fold partition, these estimates were treated as development/model-selection performance rather than independent internal validation (20). The two leading algorithms were advanced to feature reduction.

### Feature reduction and final model selection

The two leading algorithms were subjected to SHAP-guided feature reduction (21). Global SHAP rankings were obtained from models fitted on the full development cohort and subsequently reused to evaluate progressively smaller panels by five-fold cross-validation, while retaining age, sex, and eGFRcr in all candidate models. Thus, SHAP ranking was not nested within the reduction folds. Hyperparameters were held fixed during feature reduction. A reduced panel was considered near-optimal if, relative to the model-specific highest-AUPRC configuration, the decreases in AUROC and AUPRC were each ≤0.01 and the increase in Brier score was ≤0.01; the smallest qualifying panel was provisionally preferred.

An additional development-stage stability analysis compared the neighboring eight- and nine-predictor XGBoost panels after initial feature reduction, using 10 repeats of paired stratified five-fold cross-validation with identical fold assignments. This comparison was added during development rather than prespecified at study initiation, and its calculations used development-cohort data only. The final nine-predictor XGBoost model included age, sex, eGFRcr, hemoglobin, platelet count, blood urea nitrogen, albumin, alkaline phosphatase, and glucose. Feature-reduction results and the neighboring-panel comparison are provided in **Supplementary Table S4A, B**, respectively.

### External validation and model evaluation

The final nine-variable XGBoost pipeline was fitted on the complete development cohort and applied unchanged to the independent external validation cohort (22). A clinical core comparator was prespecified as logistic regression using age, sex, and eGFRcr. Additional post-review comparator analyses used logistic regression with eGFRcr alone or eGFRcr, blood urea nitrogen, and albumin; these models were developed using development data and then applied without refitting to the external cohort (Supplementary Methods 4). Model performance was assessed using AUROC, AUPRC, and Brier score. Ninety-five percent confidence intervals and paired XGBoost-versus-core differences were estimated using 2,000 patient-level bootstrap resamples, with identical resamples used for both models within each cohort. Development intervals were based on the saved pooled out-of-fold predictions and did not repeat model selection.

Calibration was evaluated using the calibration intercept, calibration slope, calibration-in-the-large, and Brier score (20, 22). Because the original external probabilities showed center-level calibration drift, a *post-hoc* five-fold cross-fitted logistic recalibration was additionally performed within the external cohort to assess local calibration. Parameters estimated in each training partition were applied only to the corresponding held-out partition; the XGBoost model itself was not retrained. This locally adapted assessment was not considered a second independent external validation. Original external probabilities remained the primary outputs for discrimination, precision-recall analysis, Brier score, decision-curve analysis, and subsequent targeted-testing analyses. Original external calibration is displayed in **Figure 1D**, **Supplementary Table S6A**, and **Supplementary Figure S3**; local cross-fitted recalibration is displayed in **Supplementary Figure S4**.

**Figure 1:**
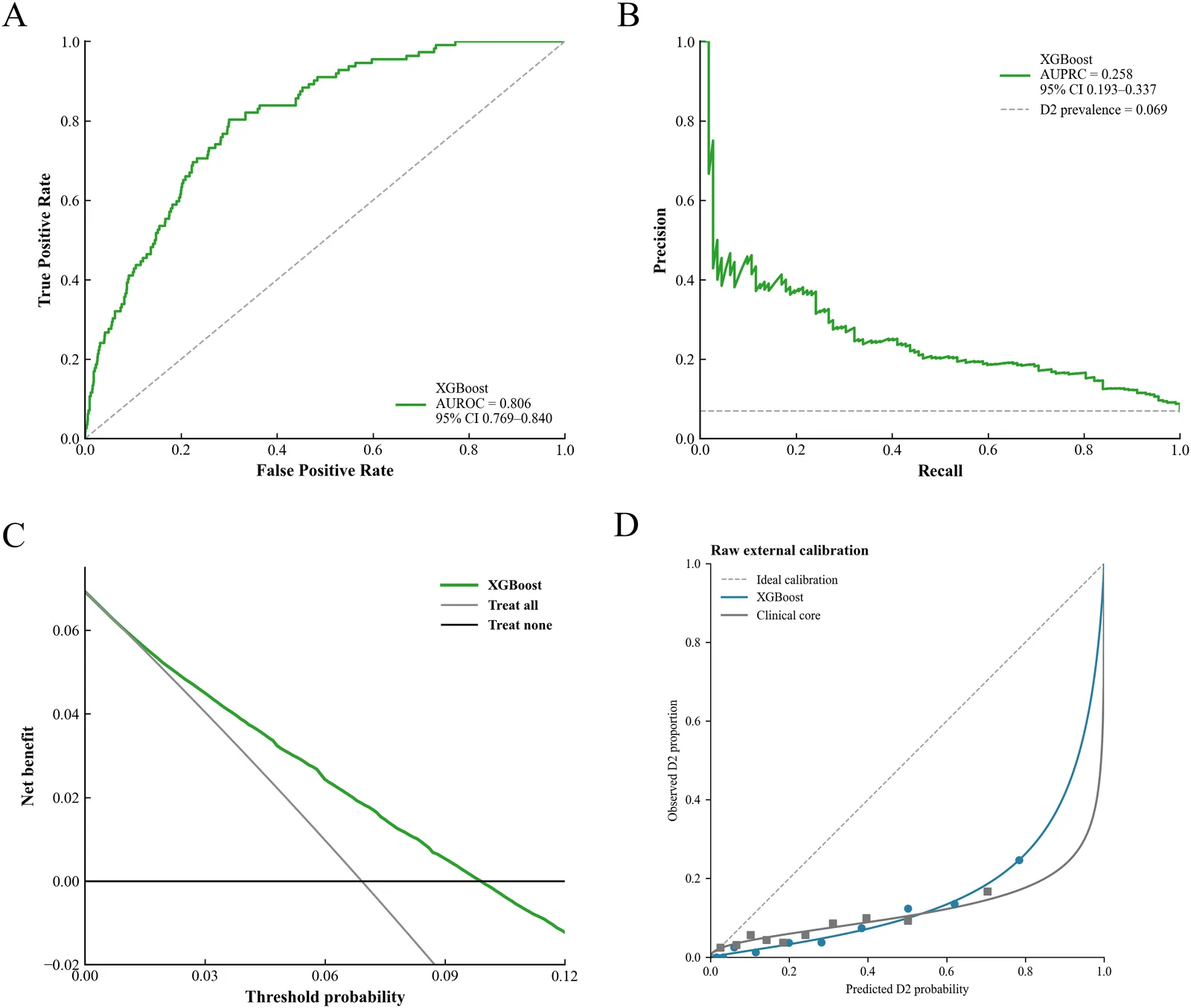
Independent external validation, decision analysis, and calibration. **(A)** ROC, **(B)** precision–recall, and **(C)** exploratory decision curves for the unchanged XGBoost model in the external cohort (n=1,619; 112 D2 events). **(D)** Original external calibration for XGBoost and the clinical core. All panels use unrecalibrated external predictions. The dashed horizontal line in B indicates D2 prevalence. In D, points show observed D2 proportions by predicted-risk decile, solid lines show logistic calibration fits, and the diagonal indicates ideal calibration; fitted curves outside observed prediction ranges are extrapolations. The 0–12% range in C is descriptive, not a validated clinical decision-threshold interval.

Decision-curve analysis was conducted as an exploratory assessment using the original external probabilities, with treat-all and treat-none as reference strategies (23). The 0–12% probability range was used for display, not as an established clinical decision-threshold interval; a wider-range and locally recalibrated sensitivity analysis is provided in **Supplementary Figure S5**.

### Model interpretation and targeted cystatin C testing

SHapley Additive exPlanations (SHAP) were used to characterize the behavior of the final XGBoost model (21). Global interpretation included mean absolute SHAP values, a beeswarm summary plot, and dependence plots for the final predictors. SHAP values were interpreted on the model-output margin scale and were used descriptively to assess feature contribution and directionality; they were not interpreted as causal effects or clinical thresholds.

To evaluate a resource-constrained testing strategy, participants were ranked by predicted D2 probability. Development ranking used pooled out-of-fold predictions, whereas external ranking used the original locked external probabilities. Prespecified cystatin C testing allocations of 10%, 20%, 30%, 40%, and 50% of each cohort were evaluated. At each allocation, we calculated the proportion of all D2 cases detected (recall), the proportion of tested participants who were D2-positive (testing yield), the number of cystatin C tests required per D2 case detected, and enrichment relative to random testing. Confidence intervals were obtained by patient-level bootstrap resampling with reranking within each resample. Post-review analyses compared the final XGBoost model with the clinical core, an eGFRcr-only logistic model, and an eGFRcr–blood urea nitrogen–albumin logistic model under identical testing allocations (**Supplementary Methods 4**). These analyses evaluated testing prioritization and did not define or optimize a diagnostic probability cutoff.

### Renal phenotype characterization and statistical analysis

For secondary phenotype characterization, the fixed top-20% XGBoost testing allocation was used to define model-positive participants. Participants were cross-classified into four groups according to observed D2 status and model selection: D2-negative/model-negative, D2-negative/model-positive, D2-positive/model-negative, and D2-positive/model-positive. The primary descriptive comparison was between D2-negative/model-positive and D2-negative/model-negative participants. Related renal phenotypes included D1, D3, eGFRcys <60 mL/min/1.73 m², eGFRcys, the eGFRcys−eGFRcr difference, and cystatin C concentration. These measures were used for phenotype characterization rather than independent disease confirmation.

Continuous variables were summarized as medians with interquartile ranges and compared using the Mann-Whitney U test. Categorical variables were summarized as counts and percentages and compared using the chi-square test or Fisher exact test, as appropriate. For the primary D2-negative group comparisons, binary phenotypes were summarized using absolute risk differences with 95% confidence intervals, with risk ratios reported secondarily; group proportions were accompanied by confidence intervals. Continuous phenotypes were summarized using differences in medians with bootstrap confidence intervals. Confidence intervals for model-performance measures and targeted-testing analyses were obtained using 2,000 patient-level bootstrap resamples as specified above. All statistical tests were two-sided. No formal *a priori* sample-size calculation was performed because this retrospective study included all available eligible records. The development and external cohorts contained 214 and 112 D2 events, respectively (23.8 and 12.4 events per final predictor); these ratios are descriptive and do not establish sample-size adequacy for XGBoost. Secondary renal-phenotype comparisons, post-review model comparisons, and sensitivity analyses were exploratory and were not adjusted for multiplicity; details are provided in **Supplementary Methods 4** and **Supplementary Tables S5**–**S7** and **S10**–**S12**. Analyses were performed using Python; environments for the original modeling and post-review analyses are documented with the corresponding analysis code. Detailed interval-estimation methods, including Newcombe and Wilson procedures, are provided in **Supplementary Methods 3**.

## Results

### Study population and baseline characteristics

The phenotype-eligible populations comprised 2,012 participants in the development source and 1,775 in the external source. After application of the prespecified modeling-eligibility rule and quality control, the final cohorts included 1,170 development participants and 1,619 independent external-validation participants (**Figure 2**). D2 was present in 214 participants (18.29%) in development and 112 (6.92%) externally. Among phenotype-eligible participants, 842 (41.8%) in development and 156 (8.8%) externally were excluded because of missing or invalid required blood-count measurements; D2 frequencies among included versus excluded participants were 18.3% versus 17.8% and 6.9% versus 7.7%, respectively (**Supplementary Tables S10A, B**).

**Figure 2:**
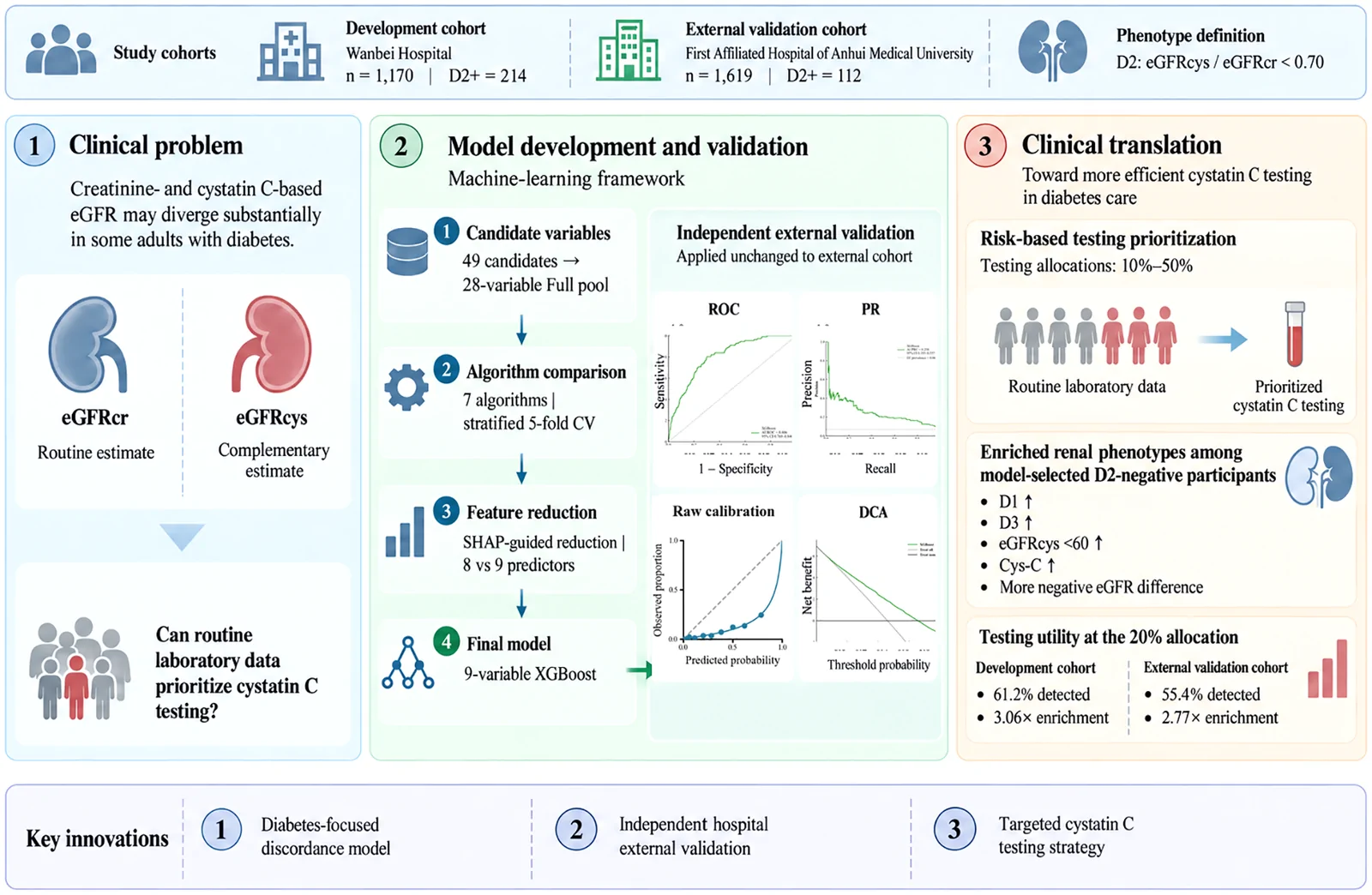
Study design and analytic workflow. D2, primary discordance phenotype defined as eGFRcys/eGFRcr <0.70; eGFRcr, creatinine-based estimated glomerular filtration rate; eGFRcys, cystatin C-based estimated glomerular filtration rate; SHAP, SHapley Additive exPlanations; XGBoost, extreme gradient boosting; ROC, receiver operating characteristic; PR, precision–recall; DCA, decision-curve analysis.

In the development cohort, D2-positive participants were older than D2-negative participants (median, 69 vs 55 years) and had lower hemoglobin (122 vs 138 g/L), albumin (40.85 vs 44.10 g/L), and eGFRcr (92.42 vs 110.03 mL/min/1.73 m²), together with higher blood urea nitrogen (7.39 vs 5.68 mmol/L); the proportion of men was similar (57.5% vs 59.4%; **Table 1**). The external validation cohort showed the same broad pattern for age, hemoglobin, albumin, and eGFRcr despite its lower D2 prevalence (**Supplementary Table S2**). Between-center distributions differed notably for eGFRcr, albumin, and glucose (standardized mean differences, 0.502, 0.527, and 0.473, respectively; **Supplementary Table S10C**).

**Table 1:** Baseline characteristics of the Wanbei development cohort.

Characteristic	D2-negative (n=956)	D2-positive (n=214)	P value
Age, years	55.0 [49.0, 61.2]	69.0 [58.0, 75.8]	<0.001
Sex (male), n (%)	568 (59.4%)	123 (57.5%)	0.602
HGB, g/L	138.00 [125.00, 149.00]	122.00 [107.25, 135.75]	<0.001
MCV, fL	90.40 [87.50, 93.00]	90.35 [87.80, 93.07]	0.678
MCHC, g/L	334.00 [327.00, 340.00]	330.00 [322.00, 336.00]	<0.001
RDW, %	12.80 [12.40, 13.20]	13.10 [12.70, 13.70]	<0.001
PLT, 10^9^/L	219.00 [181.75, 263.00]	204.50 [155.00, 265.50]	0.015
MPV, fL	10.10 [9.40, 11.10]	10.10 [9.30, 11.00]	0.975
NEUT#, 10^9^/L	3.70 [2.90, 4.76]	4.18 [3.10, 5.92]	<0.001
LYMPH#, 10^9^/L	1.80 [1.40, 2.20]	1.60 [1.20, 2.00]	<0.001
MONO#, 10^9^/L	0.37 [0.29, 0.46]	0.42 [0.32, 0.55]	<0.001
EO#, 10^9^/L	0.10 [0.06, 0.17]	0.11 [0.06, 0.18]	0.818
BASO#, 10^9^/L	0.03 [0.02, 0.04]	0.03 [0.02, 0.04]	0.973
BUN, mmol/L	5.68 [4.70, 6.97]	7.39 [5.97, 9.68]	<0.001
UA, µmol/L	286.00 [235.00, 351.00]	310.50 [260.25, 385.25]	<0.001
ALB, g/L	44.10 [41.40, 46.40]	40.85 [36.75, 44.35]	<0.001
A/G	1.87 [1.66, 2.09]	1.68 [1.41, 1.90]	<0.001
ALT, U/L	17.00 [11.00, 24.00]	13.00 [9.00, 21.00]	<0.001
AST, U/L	19.00 [15.00, 24.00]	20.00 [16.00, 26.00]	0.332
ALP, U/L	81.00 [68.25, 101.00]	86.50 [69.00, 101.00]	0.208
GGT, U/L	24.00 [17.00, 39.00]	20.00 [14.00, 31.00]	<0.001
TBIL, µmol/L	15.00 [11.00, 19.00]	12.70 [9.00, 16.00]	<0.001
Na, mmol/L	139.00 [137.00, 141.00]	139.85 [137.67, 142.00]	0.014
K, mmol/L	4.01 [3.76, 4.25]	4.05 [3.74, 4.31]	0.235
Cl, mmol/L	102.10 [100.00, 104.00]	103.80 [100.90, 106.00]	<0.001
Ca, mmol/L	2.33 [2.25, 2.41]	2.28 [2.16, 2.37]	<0.001
GLU, mmol/L	9.64 [7.32, 12.57]	7.69 [5.94, 11.05]	<0.001
eGFRcr (2021), mL/min/1.73 m²	110.03 [102.80, 117.91]	92.42 [72.03, 101.65]	<0.001
SCr, mg/dL	0.60 [0.50, 0.71]	0.78 [0.64, 1.02]	<0.001
Cystatin C, mg/L	0.71 [0.58, 0.85]	1.30 [1.16, 1.67]	<0.001
eGFRcys (2012), mL/min/1.73 m²	109.83 [94.42, 122.85]	50.37 [36.93, 61.04]	<0.001
eGFRcys − eGFRcr, mL/min/1.73 m²	-0.09 [-10.10, 8.80]	-37.64 [-44.35, -30.70]	<0.001
eGFRcys/eGFRcr	1.00 [0.90, 1.08]	0.57 [0.50, 0.65]	<0.001

Values are median [Q1, Q3] or n (%), based on available observations without imputation. Continuous variables were compared using two-sided Mann–Whitney U tests; sex was compared using the Pearson chi-square test. D2-positive indicates eGFRcys/eGFRcr <0.70. The first 28 variables constitute the Full predictor pool; the remaining rows are reference or phenotype measurements, not additional predictors. Cystatin C-derived measurements were excluded from predictors, and SCr was excluded because of structural redundancy with eGFRcr.

HGB, hemoglobin; MCV, mean corpuscular volume; MCHC, mean corpuscular hemoglobin concentration; RDW, red-cell distribution width; PLT, platelet count; MPV, mean platelet volume; NEUT/LYMPH/MONO/EO/BASO, neutrophils/lymphocytes/monocytes/eosinophils/basophils (#, absolute count); BUN, blood urea nitrogen; UA, uric acid; ALB, albumin; A/G, albumin-to-globulin ratio; ALT/AST, alanine/aspartate aminotransferase; ALP, alkaline phosphatase; GGT, gamma-glutamyl transferase; TBIL, total bilirubin; Na, sodium; K, potassium; Cl, chloride; Ca, calcium; GLU, glucose; SCr, serum creatinine; eGFRcr/eGFRcys, creatinine-/cystatin C-based estimated glomerular filtration rate.

### Model development and final predictor selection

Among the seven algorithms evaluated using the identical 28-variable Full feature pool, XGBoost, random forest, and LightGBM showed the strongest development performance (**Figure 3A**; **Supplementary Table S3**). XGBoost achieved an AUROC of 0.854, AUPRC of 0.584, and Brier score of 0.106; the corresponding values were 0.850, 0.574, and 0.107 for random forest and 0.854, 0.565, and 0.108 for LightGBM. Because AUPRC was the prespecified primary model-selection metric, XGBoost and random forest were advanced to SHAP-guided feature reduction (**Figures 3B, C**).

**Figure 3:**
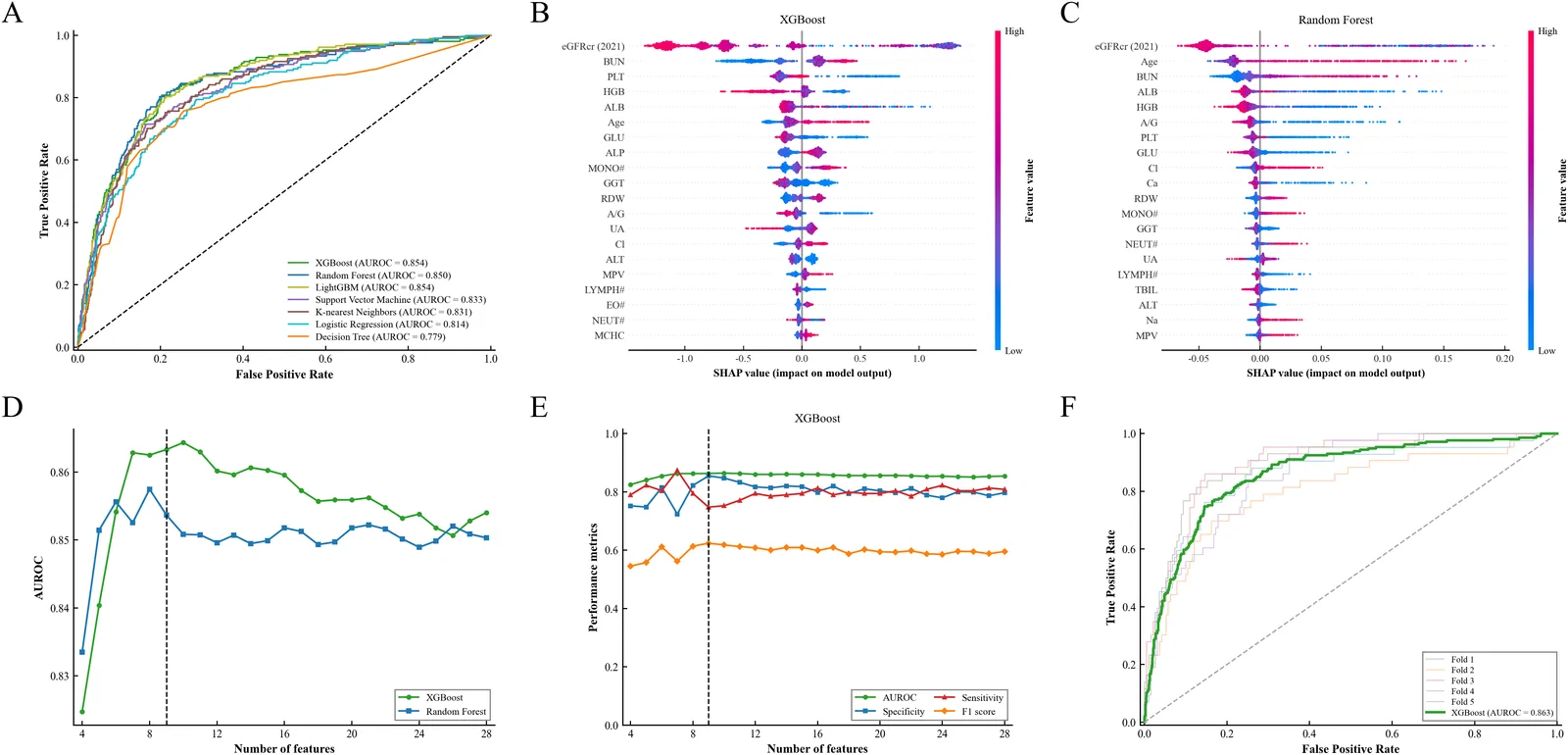
Model development and feature reduction. **(A)** Receiver operating characteristic (ROC) curves for seven algorithms using the 28-variable Full pool. **(B–C)** SHAP beeswarm summaries for the Full-pool XGBoost and random forest candidates; model-output scales differ between algorithms. **(D)** AUROC by predictor count. **(E)** XGBoost AUROC, sensitivity, specificity, and F1 score by predictor count. Classification metrics in E use the pooled development out-of-fold Youden J threshold determined separately at each predictor count and were not used for feature selection. **(F)** Final XGBoost development ROC, showing individual fold curves and the pooled OOF curve. Dashed vertical lines in D–E mark nine predictors. Development estimates reflect model selection, not independent validation. SHAP, SHapley Additive exPlanations; OOF, out-of-fold; AUROC, area under the ROC curve.

Feature-count reduction showed a broad performance plateau for both candidate models (**Figure 3D-E**; **Supplementary Table S4A**). The smallest provisionally near-optimal panels contained eight predictors for XGBoost and 11 for random forest. XGBoost remained the preferred algorithm under the prespecified selection hierarchy. Because the neighboring eight- and nine-predictor XGBoost panels performed similarly in the initial analysis, they were compared across 10 repeats of paired stratified five-fold cross-validation. The nine-predictor panel improved AUROC in all 10 repeats, AUPRC in 9 of 10, and Brier score in 7 of 10; mean differences for nine versus eight predictors were +0.0035 for AUROC, + 0.0063 for AUPRC, and -0.0004 for Brier score. These results supported retention of alkaline phosphatase as the ninth predictor.

The final XGBoost model therefore included age, sex, eGFRcr, hemoglobin, platelet count, blood urea nitrogen, albumin, alkaline phosphatase, and glucose. Using pooled out-of-fold predictions in the development cohort, the final model achieved an AUROC of 0.863 (95% CI, 0.833-0.889), an AUPRC of 0.600 (95% CI, 0.532-0.668), and a Brier score of 0.102 (95% CI, 0.091-0.115) (**Figure 3F**). The corresponding clinical-core values were 0.791, 0.461, and 0.122, yielding paired differences of +0.073 (95% CI, 0.044-0.103) in AUROC, + 0.139 (95% CI, 0.073-0.201) in AUPRC, and -0.019 (95% CI, -0.028 to -0.011) in Brier score (**Supplementary Table S5**). Model fitting, tuning, SHAP ranking, and reduced-panel evaluation used development-cohort data, while external predictor metadata informed only feasibility and harmonization review.

### Independent external validation

In the independent external validation cohort, the final nine-variable XGBoost model retained discrimination, with an AUROC of 0.806 (95% CI, 0.769-0.840) and an AUPRC of 0.258 (95% CI, 0.193-0.337) (**Figures 1A, B**). The clinical core achieved an AUROC of 0.661 (95% CI, 0.605-0.715) and an AUPRC of 0.126 (95% CI, 0.096-0.176). Corresponding paired XGBoost-minus-core differences were +0.145 (95% CI, 0.104-0.187) for AUROC and +0.132 (95% CI, 0.067-0.198) for AUPRC. The raw external Brier score was 0.144 (95% CI, 0.135-0.153), compared with 0.130 (95% CI, 0.122-0.140) for the clinical core. The lower external AUPRC relative to development (0.258 vs 0.600) occurred alongside a lower D2 prevalence (6.9% vs 18.3%). At an external ROC operating point achieving at least 80% sensitivity, specificity was 70.0% for XGBoost versus 35.4% for the clinical core; these cohort-specific descriptive operating points are detailed in **Supplementary Table S5D**.

Decision-curve analysis based on the original external probabilities did not show consistently positive net benefit over the exploratory 0–12% threshold-probability display range (**Figure 1C**). Net benefit was −0.0007 at a 10% threshold and −0.0122 at 12% (**Supplementary Table S6C**); locally recalibrated and wider-range sensitivity results are shown in **Supplementary Figure S5**.

Although ranking performance transported externally, the original probabilities overestimated absolute D2 risk: the mean predicted probability was 29.9% compared with an observed prevalence of 6.9%, with a calibration intercept of -2.209, slope of 0.848, and calibration-in-the-large of -2.250 (**Figure 1D**; **Supplementary Table S6A**; **Supplementary Figure S3**). In the *post-hoc* five-fold cross-fitted local recalibration assessment, the calibration intercept was -0.039, the slope was 0.981, and the Brier score was 0.058 (**Supplementary Figure S4**; **Supplementary Tables S6A, B**). This locally adapted assessment did not retrain the XGBoost model or constitute a second independent external validation; the primary ROC, precision–recall, decision-curve, and targeted-testing results remained based on the original locked external probabilities.

### Model interpretation

Global SHAP analysis of the final nine-variable XGBoost model identified eGFRcr as the largest contributor to model output, with a mean absolute SHAP value of 0.887, followed by blood urea nitrogen (0.354), albumin (0.292), platelet count (0.289), hemoglobin (0.244), glucose (0.203), alkaline phosphatase (0.193), age (0.186), and sex (0.077) (**Figures 4A, B**). Dependence plots showed marked nonlinear behavior for several predictors (**Figure 4C**). Higher eGFRcr values generally shifted model output toward D2-negative predictions, whereas higher blood urea nitrogen tended to shift predictions toward D2-positive values before reaching a plateau. Lower albumin and lower platelet counts were generally associated with positive SHAP contributions, while higher hemoglobin values tended to shift model output toward D2-negative predictions. Age showed an overall positive contribution at older values. These patterns describe how the fitted model used the predictors and were not interpreted as causal effects or clinical thresholds.

**Figure 4:**
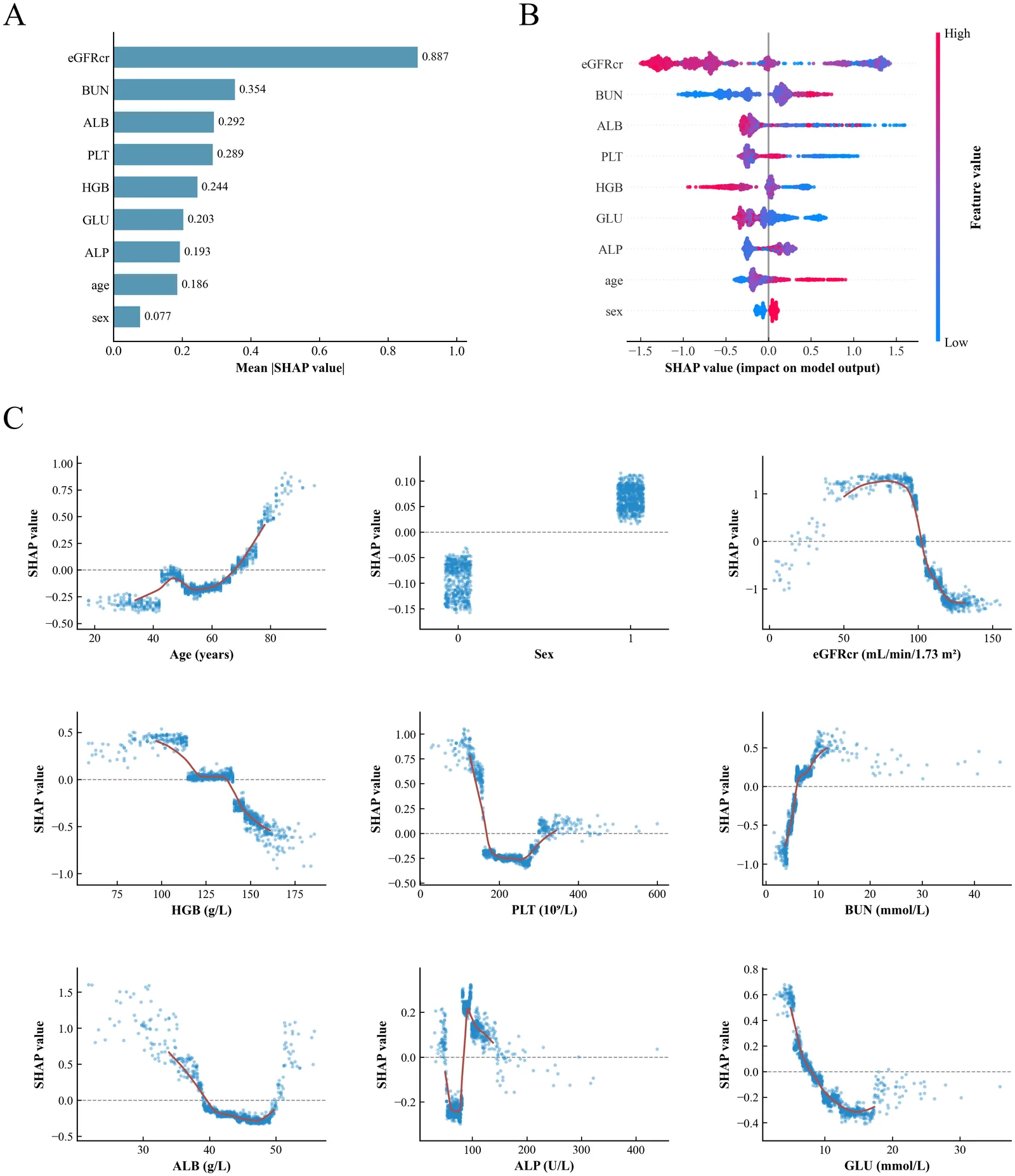
Interpretation of the final nine-predictor XGBoost model. **(A)** Mean absolute SHAP contributions, **(B)** beeswarm summary, and **(C)** dependence plots for all nine predictors. SHAP values are on the raw-margin/log-odds scale. Point color in B represents predictor value; sex is coded 0 for female and 1 for male. Lines in the continuous-predictor dependence plots summarize the plotted relationships; dashed horizontal lines mark zero contribution. These patterns describe model output, not causal effects or diagnostic thresholds. eGFRcr is also a component of the ratio defining D2. BUN, blood urea nitrogen; ALB, albumin; PLT, platelet count; HGB, hemoglobin; GLU, glucose; ALP, alkaline phosphatase.

### Targeted cystatin C testing

Risk-based prioritization substantially enriched D2 detection under fixed cystatin C testing allocations (**Figure 5**; **Supplementary Table S7**). At the prespecified 20% testing allocation, 234 development participants were selected and 131 of 214 D2 cases were identified, corresponding to a recall of 61.21% (95% CI, 55.71-67.49), a testing yield of 55.98% (95% CI, 49.15-63.25), and 3.06-fold enrichment over random testing (95% CI, 2.79-3.37). This corresponded to 1.79 cystatin C tests per D2 case detected (95% CI, 1.58-2.03).

**Figure 5:**
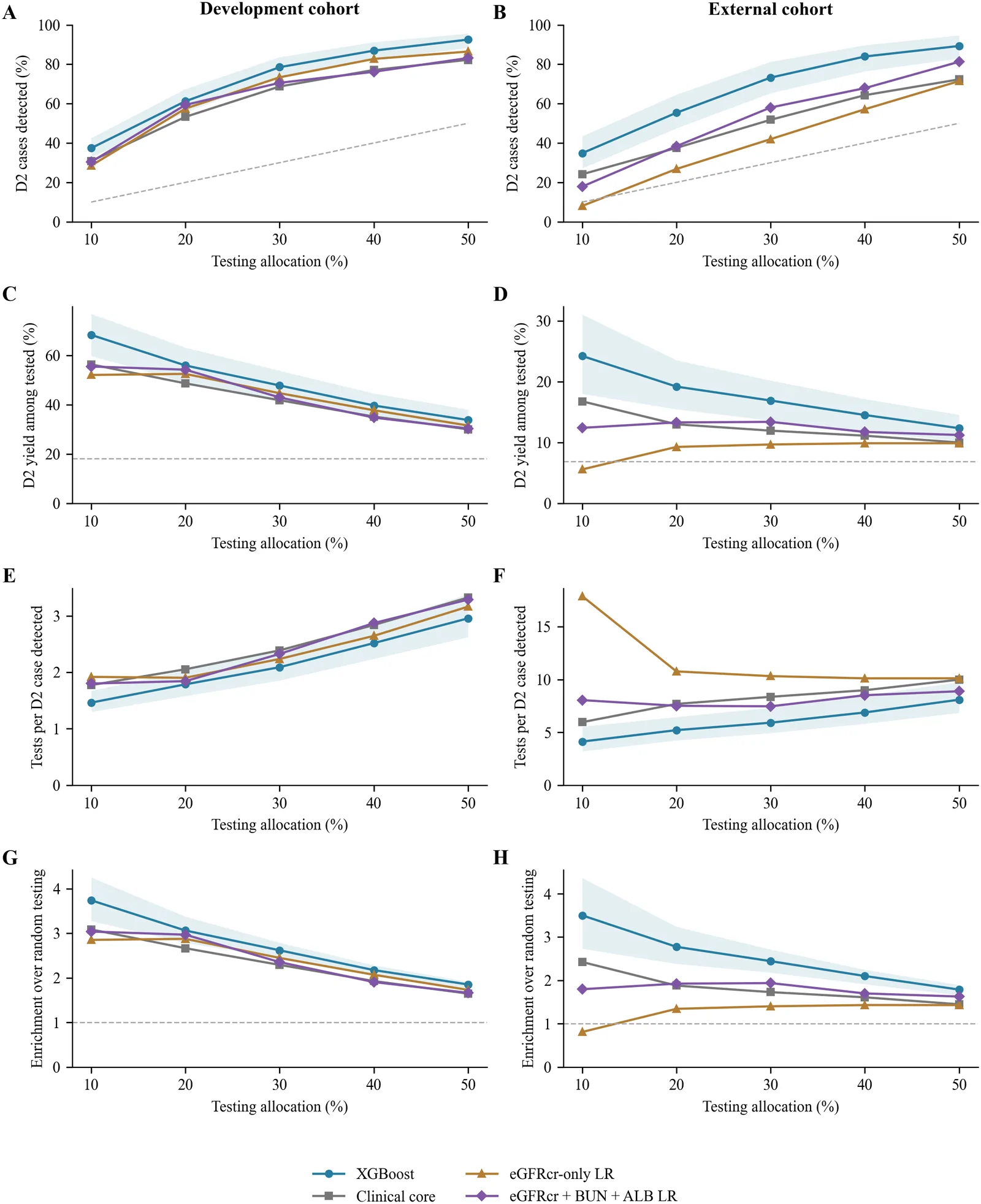
Targeted cystatin C testing across fixed allocations. **(A, B)** Percentage of D2 cases detected (recall), **(C, D)** yield among participants selected for testing, **(E, F)** tests per detected D2 case, and **(G, H)** enrichment over random testing across 10–50% testing allocations. Left panels show development out-of-fold estimates; right panels show independent external validation. Curves compare XGBoost, the clinical core, eGFRcr-only logistic regression, and eGFRcr + BUN + ALB logistic regression. Shading represents XGBoost pointwise 95% bootstrap confidence intervals; dashed lines show random-testing benchmarks where applicable. Testing allocations are ranking-based, not probability cutoffs. eGFRcr, creatinine-based estimated glomerular filtration rate; BUN, blood urea nitrogen; ALB, albumin.

In the independent external validation cohort, testing the highest-ranked 20% selected 323 participants and identified 62 of 112 D2 cases, yielding a recall of 55.36% (95% CI, 47.52-64.65), a testing yield of 19.20% (95% CI, 15.48-23.53), and 2.77-fold enrichment over random testing (95% CI, 2.38-3.24). The corresponding number of cystatin C tests per D2 case detected was 5.21 (95% CI, 4.25-6.46). Across testing allocations from 10% to 50%, D2 recall increased from 37.38% to 92.52% in development and from 34.82% to 89.29% externally, while testing yield and enrichment progressively declined, illustrating the expected trade-off between case capture and testing efficiency. At the same 20% external allocation, the clinical core, eGFRcr-only logistic model, and three-variable logistic model detected 42 (37.5%), 30 (26.8%), and 43 (38.4%) D2 cases, respectively, versus 62 (55.4%) with XGBoost (**Figure 5**; **Supplementary Table S7B**). The paired XGBoost-minus-three-variable recall difference was +17.0 percentage points (95% CI, 8.6–27.2) externally but only +1.9 (95% CI, −2.7 to 6.9) in development (**Supplementary Table S7C**).

### Renal phenotype characteristics of model-selected participants

Using the fixed top-20% testing allocation, participants were classified into four groups according to observed D2 status and model selection (**Figure 6A**). Among D2-negative participants, those selected by the model showed consistently higher prevalence of related cystatin C-based renal phenotypes in both cohorts. In development, D1 was present in 28.16% of model-positive versus 14.65% of model-negative participants (absolute risk difference, +13.50 percentage points; 95% CI, 5.32-23.11), D3 in 10.53% versus 0.24% (+10.29 percentage points; 95% CI, 5.15-19.18), and eGFRcys <60 mL/min/1.73 m² in 31.07% versus 2.11% (+28.96 percentage points; 95% CI, 20.75-38.47). In the external cohort, corresponding prevalences were 19.92% versus 8.03% for D1 (+11.90 percentage points; 95% CI, 7.21-17.34), 13.09% versus 1.65% for D3 (+11.44 percentage points; 95% CI, 7.27-17.00), and 32.57% versus 12.12% for eGFRcys <60 mL/min/1.73 m² (+20.45 percentage points; 95% CI, 14.72-26.59) (**Figures 6B–D**; **Supplementary Tables S8**, **S9**).

**Figure 6:**
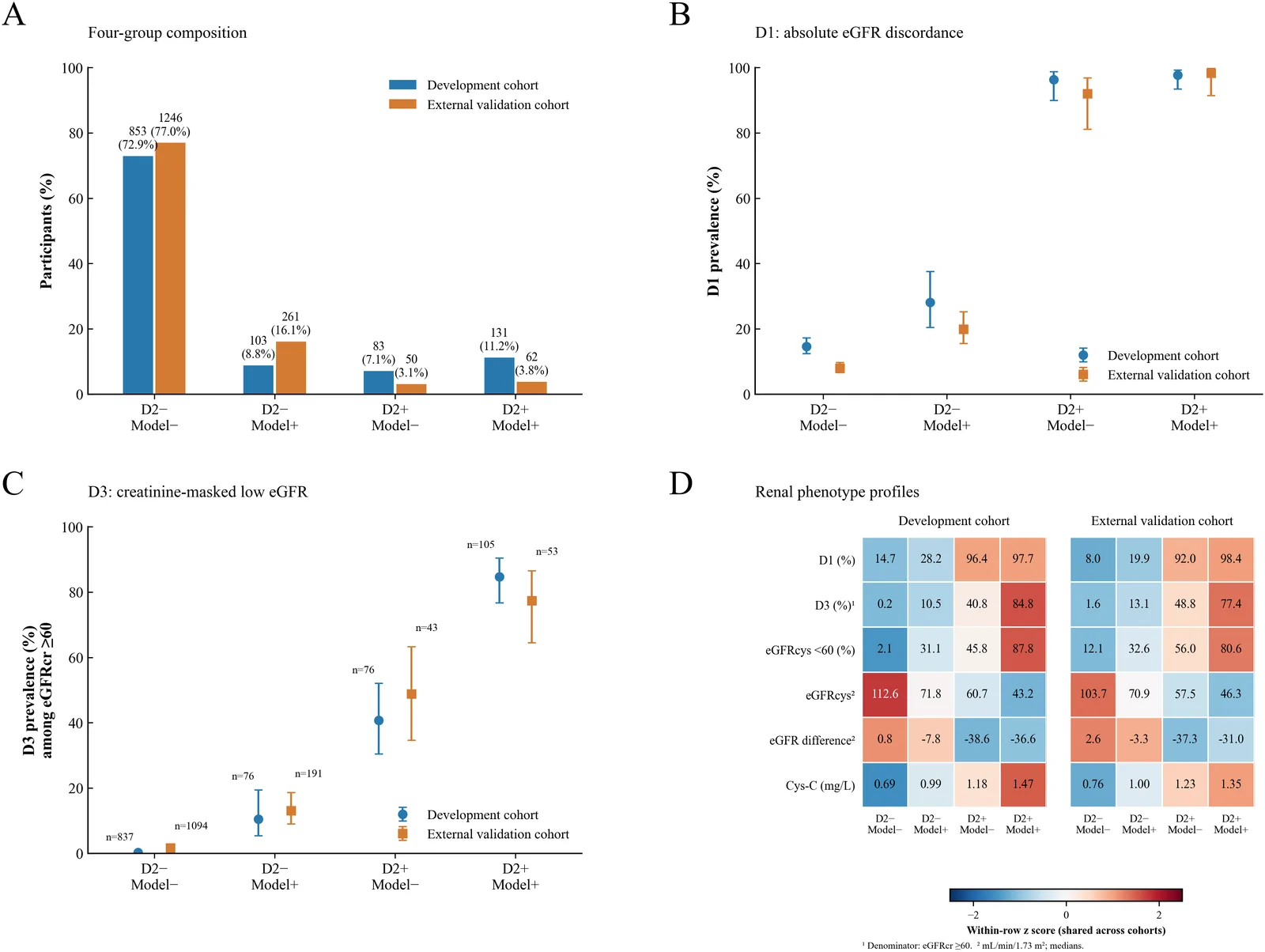
Renal phenotype profiles by observed D2 and model-selected testing status. Model-positive denotes the top 20% selected by XGBoost. **(A)** Four groups defined by D2 and model-selection status. **(B)** D1 prevalence (eGFRcys − eGFRcr ≤−15 mL/min/1.73 m²). **(C)** D3 prevalence (eGFRcys <60 among participants with eGFRcr ≥60 mL/min/1.73 m²); labels indicate eligible group sizes. Error bars in B–C represent Wilson 95% confidence intervals. **(D)** Related renal measures, with values showing prevalences or medians and colors indicating within-row standardized values across cohorts. The eGFR difference is eGFRcys − eGFRcr. eGFRcr/eGFRcys, creatinine-/cystatin C-based estimated glomerular filtration rates.

Continuous renal measures showed the same pattern. Among D2-negative participants, the median difference in eGFRcys (model-positive minus model-negative) was −40.80 mL/min/1.73 m² in development (95% CI, −45.08 to −34.33) and −32.78 mL/min/1.73 m² externally (95% CI, −35.77 to −29.38). The corresponding median differences in eGFRcys−eGFRcr were −8.53 and −5.92 mL/min/1.73 m², while cystatin C concentrations were higher by 0.30 mg/L and 0.24 mg/L, respectively. These findings indicate that model-selected D2-negative participants were enriched for related cystatin C-based renal phenotypes, without implying independent confirmation of kidney disease. In additional post-review analyses, complete-case evaluation gave an external AUROC of 0.812, and a fixed-structure mean-imputation sensitivity model gave an external AUROC of 0.805; the original frozen D2 score identified D1 with AUROC values of 0.778 in development and 0.731 externally (**Supplementary Table S11**). Among selectively retested participants with another valid paired measurement 30–365 days later, D2 status agreement was 66.1% (n=59; κ=0.175) in development and 86.8% (n=53; κ=0.457) externally (**Supplementary Table S12**). These analyses did not establish phenotype chronicity or measured-GFR accuracy.

## Discussion

In this two-center study of adults with diabetes, a nine-predictor model based on routinely available demographic and laboratory data showed better discrimination and precision-recall performance for large negative eGFR discordance than an age-sex-eGFRcr clinical core in both the development and independent external validation cohorts. More importantly, model ranking translated into a practical testing-prioritization strategy: restricting cystatin C measurement to the highest-ranked 20% identified 61.2% of D2 cases in development and 55.4% externally. Among participants who did not meet the primary D2 definition, those selected by the model were also enriched for related cystatin C-based renal phenotypes in both cohorts. Together, these findings support risk-guided prioritization of cystatin C measurement when universal testing is impractical, while retaining cystatin C measurement as the required measurement for ascertainment of the discordance phenotype.

Large negative discordance between creatinine- and cystatin C-based eGFR is increasingly recognized as clinically relevant. Recent individual-level and aggregate meta-analyses have associated substantially lower eGFRcys than eGFRcr with mortality, cardiovascular events, heart failure, and kidney failure (5, 6), while routine-care data show similar associations across multiple adverse outcomes (7). Diabetes-specific studies have linked differences between the two estimates with microvascular complications, incident albuminuria, mortality, and cardiovascular events (8, 9, 24), and a 2025 UK Biobank analysis further associated lower relative eGFRcys with higher all-cause mortality (25). Recent reviews have also emphasized broader clinical availability of cystatin C and its particular relevance to kidney-function assessment in diabetes (26–28). Additional diabetes cohorts have linked eGFR discordance with diabetes-related multimorbidity and adverse renal or mortality outcomes (29, 30). Measured-GFR comparisons indicate that neither single-marker estimate should automatically be assumed to be closer to true GFR when the estimates disagree; combined-marker equations can improve accuracy, although performance varies with the equation and population (4, 31, 32). Prediction-guided cystatin C testing, however, is not a new concept: Peralta et al. externally validated a clinical score for occult cystatin C-defined low eGFR and evaluated case detection across different testing proportions (11), and Chen et al. characterized and modeled eGFR differences in the UK Biobank (12). More broadly, recent machine-learning studies in diabetes have used routine clinical and laboratory data for DKD identification and risk prediction (13–16). The present study adds evidence for a distinct target phenotype: substantial relative creatinine-cystatin C discordance in adults with diabetes, evaluated with an independent hospital cohort and identical fixed testing allocations in development and external validation.

The model appears to integrate both the structural information contained in eGFRcr and additional signals from routine laboratory measurements. eGFRcr was the dominant SHAP contributor, which is expected because it is mathematically embedded in the D2 definition and should not be interpreted as an independent biological risk factor. Blood urea nitrogen, albumin, platelet count, hemoglobin, glucose, alkaline phosphatase, age, and sex contributed additional, often nonlinear, information to model output. These SHAP patterns describe how the fitted model generated its predictions rather than causal relationships. Recent measured-GFR and CKD cohort studies likewise suggest that creatinine-cystatin C discordance has complex determinants and should not be reduced to a single biological mechanism (32, 33). The phenotype analysis provides complementary context: among participants who did not meet the D2 definition, model-selected individuals had substantially lower eGFRcys, more negative eGFRcys-eGFRcr differences, higher cystatin C concentrations, and greater prevalence of D1, D3, and eGFRcys <60 mL/min/1.73 m² in both cohorts. This consistency suggests that the model ranking captured a broader continuum of cystatin C-related renal discordance, although these related measures share cystatin C and therefore do not constitute independent confirmation of kidney disease.

The principal clinical application of the model is therefore testing prioritization rather than diagnosis. When universal cystatin C measurement is impractical, ranking based on routine laboratory data may help identify patients with a higher likelihood of D2 for additional testing. At the illustrative 20% testing allocation, the model identified 61.2% of D2 cases in development and 55.4% in external validation, with 3.06-fold and 2.77-fold enrichment over random testing, respectively. The corresponding numbers of cystatin C tests required per D2 case detected were 1.79 and 5.21. At the same 20% allocation externally, XGBoost identified 62 D2 cases versus 42 with the clinical core, 30 with eGFRcr-only logistic regression, and 43 with an eGFRcr + BUN + ALB logistic model; the paired recall difference versus the latter was +17.0 percentage points (95% CI, 8.6–27.2). The corresponding difference in development was smaller and uncertain (+1.9 percentage points; 95% CI, −2.7 to 6.9). Performance across the 10%-50% allocations also demonstrated the expected resource trade-off: increasing the proportion tested improved case capture but progressively reduced testing yield and enrichment. This framework allows testing intensity to be adapted to local laboratory capacity rather than imposing a single probability threshold. The potential clinical consequences of eGFR discordance extend beyond disease classification; in hospitalized patients, discordance may alter dosing decisions for renally eliminated medications (34), and institutional implementation studies show that cystatin C-guided kidney-function assessment requires coordinated laboratory and clinical workflows (35). Interpretation nevertheless requires clinical context, particularly because cystatin C is affected by non-GFR determinants and may contribute to CKD overclassification in some older adults (36). Other recent studies support the prognostic value of cystatin C in populations with prediabetes or diabetes (37) and show that cystatin C- and creatinine-based kidney-function estimates can stratify cardiovascular risk differently across populations (38). Implementation will therefore depend on local assay availability, standardization, and laboratory practice (10, 28, 39, 40). Following cystatin C measurement, substantial discordance may prompt clinician-led assessment of combined creatinine–cystatin C eGFR and review of kidney-function-dependent medication dosing or the need for further renal evaluation (2, 4, 34). Model rank or D2 status alone does not mandate dose adjustment or referral. The present analysis was retrospective and does not establish cost-effectiveness, improved clinical outcomes, or the optimal testing allocation for a particular health system.

The external validation findings also distinguish transport of patient ranking from transport of absolute predicted probability. Because average precision depends on outcome prevalence, the lower external D2 frequency may partly explain the decrease in AUPRC, although differences in case mix and discrimination may also contribute. Despite a marked difference in D2 prevalence between development and external validation (18.3% vs 6.9%), the model retained useful discrimination and continued to enrich D2 cases under the same rank-based testing allocations. In contrast, the original external probabilities systematically overestimated absolute D2 risk, with a mean predicted probability of 29.9% compared with an observed prevalence of 6.9%. The original external Brier score was 0.144 for XGBoost versus 0.130 for the clinical core, indicating that better discrimination did not translate into lower overall probability error. *Post-hoc* cross-fitted logistic recalibration substantially improved local calibration without retraining the XGBoost model. Because this recalibration was performed within the external cohort, it should be regarded as a local calibration assessment rather than additional independent validation. Decision-curve analysis using original external probabilities did not show consistently positive net benefit throughout the exploratory 0–12% display range, which lacked an established clinical threshold rationale. These findings suggest that the current model is better supported for risk ranking and testing prioritization than for direct transfer of uncalibrated absolute probabilities across centers; local calibration would be required if probability-based decision thresholds were to be used.

Several limitations should be considered. First, the study was retrospective, and hyperparameter selection and pooled out-of-fold evaluation in the development cohort used the same five-fold partition; development estimates therefore represent model-selection performance rather than independent internal validation. The independent external cohort provides the principal evidence for generalization. Global SHAP rankings derived from the full development cohort were also reused during reduced-panel cross-validation, adding potential feature-selection optimism to development estimates. Second, D2 and the related renal phenotypes were defined from a single paired creatinine-cystatin C measurement and do not establish chronicity-confirmed CKD. Measured GFR was unavailable, so the study evaluates discordance between filtration estimates rather than determining which estimate more accurately reflects true GFR. In selectively retested subsets, D2 classification agreed after 30–365 days in 66.1% of development participants (n=59) and 86.8% of external participants (n=53), but these data cannot establish phenotype persistence in the full cohorts. Third, eGFRcr is mathematically embedded in the D2 definition and was also included as a predictor, creating an expected structural association; comparison with the age-sex-eGFRcr clinical core was therefore important for assessing the incremental information provided by the full model. Fourth, participants had already undergone paired creatinine–cystatin C testing and were required to have measured WBC, HGB, and PLT, which may limit applicability to unselected adults with diabetes. Cystatin C testing indications were not reliably available, and differences in case mix and testing practices may have contributed to the inter-center prevalence shift. Diabetes type could not be reliably distinguished, and evaluation was limited to two Chinese centers; broader transportability remains to be established. Finally, the testing-allocation analysis was retrospective and did not assess downstream changes in clinical management, patient outcomes, or cost-effectiveness. Nevertheless, the combination of independent external validation and consistent enrichment under fixed testing allocations supports prospective evaluation of routine-data-guided cystatin C testing in adults with diabetes.

## Conclusion

In adults with diabetes who underwent paired creatinine and cystatin C testing, a nine-predictor model based on routinely available demographic and laboratory data identified large negative cystatin C-creatinine eGFR discordance with useful discrimination in an independent external cohort. Risk-based prioritization substantially enriched D2 case detection under limited cystatin C testing allocations, supporting further evaluation of targeted cystatin C testing when universal measurement is impractical. Prospective evaluation is required before clinical implementation, particularly to establish locally appropriate calibration, testing allocation, and downstream clinical benefit.

## Data Availability

The raw data supporting the conclusions of this article will be made available by the authors, without undue reservation.

## References

[1] American Diabetes Association Professional Practice Committee for Diabetes . 11. Chronic kidney disease and risk management: Standards of care in diabetes-2026. Diabetes Care. (2026) 49:S246–60. doi: 10.2337/dc26-S011 41358881 PMC12690176

[2] Kidney Disease: Improving Global Outcomes (KDIGO) CKD Work Group . KDIGO 2024 clinical practice guideline for the evaluation and management of chronic kidney disease. Kidney Int. (2024) 105:S117–314. doi: 10.1016/j.kint.2023.10.018 38490803

[3] Stehle T Delanaye P . Which is the best glomerular filtration marker: creatinine, cystatin C or both? Eur J Clin Invest. (2024) 54:e14278. doi: 10.1111/eci.14278 38949475

[4] Fu EL Levey AS Coresh J Elinder CG Rotmans JI Dekker FW . Accuracy of GFR estimating equations in patients with discordances between creatinine and cystatin C-based estimations. J Am Soc Nephrol. (2023) 34:1241–51. doi: 10.1681/ASN.0000000000000128 36995139 PMC10356168

[5] Estrella MM Ballew SH Sang Y Grams ME Coresh J Surapaneni A . Discordance in creatinine- and cystatin C-based eGFR and clinical outcomes: A meta-analysis. JAMA. (2025) 334:1915–26. doi: 10.1001/jama.2025.17578 41202182 PMC12595547

[6] Liu Q Welsh P Celis-Morales C Ho FK Lees JS Mark PB . Discordance between cystatin C-based and creatinine-based estimated glomerular filtration rate and health outcomes in adults: a systematic review and meta-analysis. Clin Kidney J. (2025) 18:sfaf003. doi: 10.1093/ckj/sfaf003 40235956 PMC11997436

[7] Carrero JJ Fu EL Sang Y Ballew S Evans M Elinder CG . Discordances between creatinine- and cystatin C-based estimated GFR and adverse clinical outcomes in routine clinical practice. Am J Kidney Dis. (2023) 82:534–42. doi: 10.1053/j.ajkd.2023.04.002 37354936

[8] He D Gao B Wang J Yang C Zhao MH Zhang L . The difference between cystatin C- and creatinine-based estimated glomerular filtration rate and risk of diabetic microvascular complications among adults with diabetes: a population-based cohort study. Diabetes Care. (2024) 47:873–80. doi: 10.2337/dc23-2364 38470988 PMC11043223

[9] Harjutsalo V Thorn LM Groop PH . Comparison of serum creatinine- and cystatin C-based eGFR at baseline and their prediction of incident moderate albuminuria in individuals with type 1 diabetes. Diabetes Care. (2025) 48:1204–12. doi: 10.2337/dc24-2519 40173093

[10] Awdishu L Karger AB Shlipak MG Estrella MM . Use of cystatin C in tandem with creatinine to improve GFR estimation and clinical decision making. J Am Soc Nephrol. (2026) 37:2042–54. doi: 10.1681/ASN.0000001201 42424121 PMC13567858

[11] Peralta CA Muntner P Scherzer R Judd S Cushman M Shlipak MG . A risk score to guide cystatin C testing to detect occult-reduced estimated glomerular filtration rate. Am J Nephrol. (2015) 42:141–7. doi: 10.1159/000439231 26381887 PMC4589276

[12] Chen DC Lu K Scherzer R Lees JS Rutherford E Mark PB . Cystatin C- and creatinine-based estimated GFR differences: Prevalence and predictors in the UK Biobank. Kidney Med. (2024) 6:100796. doi: 10.1016/j.xkme.2024.100796 38567244 PMC10986041

[13] Zhang M Zhu S Hu D Fu W Hu H Xu Y . Developing and validating a clinlabomics-based machine-learning model for early detection of occult diabetic kidney disease: implications for primary care screening. Front Endocrinol (Lausanne). (2026) 17:1835866. doi: 10.3389/fendo.2026.1835866 42125236 PMC13158108

[14] Yong W Peng DD Ye K Gao JJ Cao RX . Machine learning model based on routine blood and biochemical parameters for early diagnosis of diabetic kidney disease. Front Endocrinol (Lausanne). (2026) 17:1720574. doi: 10.3389/fendo.2026.1720574 41685239 PMC12890677

[15] Li Y Jin N Zhan Q Huang Y Sun A Yin F . Machine learning-based risk predictive models for diabetic kidney disease in type 2 diabetes mellitus patients: a systematic review and meta-analysis. Front Endocrinol (Lausanne). (2025) 16:1495306. doi: 10.3389/fendo.2025.1495306 40099258 PMC11911190

[16] Zheng K Liu L You J Su J Li L Chen J . Development of an explainable machine learning model for predicting the occurrence of advanced diabetic kidney disease. Front Endocrinol (Lausanne). (2026) 17:1722013. doi: 10.3389/fendo.2026.1722013 42006264 PMC13082932

[17] Collins GS Moons KGM Dhiman P Riley RD Beam AL Van Calster B . TRIPOD+AI statement: updated guidance for reporting clinical prediction models that use regression or machine learning methods. BMJ. (2024) 385:e078378. doi: 10.1136/bmj-2023-078378 38626948 PMC11019967

[18] Inker LA Eneanya ND Coresh J Tighiouart H Wang D Sang Y . New creatinine- and cystatin C-based equations to estimate GFR without race. N Engl J Med. (2021) 385:1737–49. doi: 10.1056/NEJMoa2102953 34554658 PMC8822996

[19] Inker LA Schmid CH Tighiouart H Eckfeldt JH Feldman HI Greene T . Estimating glomerular filtration rate from serum creatinine and cystatin C. N Engl J Med. (2012) 367:20–9. doi: 10.1056/NEJMoa1114248 22762315 PMC4398023

[20] Collins GS Dhiman P Ma J Schlussel MM Archer L Van Calster B . Evaluation of clinical prediction models (part 1): from development to external validation. BMJ. (2024) 384:e074819. doi: 10.1136/bmj-2023-074819 38191193 PMC10772854

[21] Lundberg SM Erion G Chen H DeGrave A Prutkin JM Nair B . From local explanations to global understanding with explainable AI for trees. Nat Mach Intell. (2020) 2:56–67. doi: 10.1038/s42256-019-0138-9 32607472 PMC7326367

[22] Riley RD Archer L Snell KIE Ensor J Dhiman P Martin GP . Evaluation of clinical prediction models (part 2): how to undertake an external validation study. BMJ. (2024) 384:e074820. doi: 10.1136/bmj-2023-074820 38224968 PMC10788734

[23] Vickers AJ Elkin EB . Decision curve analysis: a novel method for evaluating prediction models. Med Decis Making. (2006) 26:565–74. doi: 10.1177/0272989X06295361 17099194 PMC2577036

[24] He D Gao B Wang J Yang C Wu S Chen S . Diabetes mellitus: association of cystatin C- versus creatinine-based estimated glomerular filtration rate with mortality and cardiovascular events. Nephrol Dial Transplant. (2024) 39:1322–32. doi: 10.1093/ndt/gfae011 38317440

[25] Liu Q Celis-Morales C Lees JS Sattar N Ho FK Pell JP . Discordance between cystatin C-based and creatinine-based estimated glomerular filtration rate and mortality in the general population. Clin Chem. (2025) 71:858–69. doi: 10.1093/clinchem/hvaf063 40464748

[26] Lees JS Fabian J Shlipak MG . Cystatin C should be routinely available for estimating kidney function. Curr Opin Nephrol Hypertens. (2024) 33:337–43. doi: 10.1097/MNH.0000000000000980 38411195

[27] Mende CW Bloomgarden Z . Measurement of renal function: Should cystatin C be more widely used for people with diabetes? J Diabetes. (2024) 16:e13534. doi: 10.1111/1753-0407.13534 38282206 PMC10822779

[28] Karger AB Shlipak MG . Glomerular filtration rate (GFR) estimation with cystatin C-past, present, and future. Clin Chem. (2025) 71:743–51. doi: 10.1093/clinchem/hvae226 39902788

[29] Chen F Zhang Y Gao D Zhang Y Yi S Guo J . Difference between cystatin C- and creatinine-based estimated glomerular filtration rate and risk of diabetes-related multimorbidity among adults with diabetes. Diabetes Res Clin Pract. (2025) 227:112419. doi: 10.1016/j.diabres.2025.112419 40812429

[30] Gohda T Kamei N Tanaka M Furuhashi M Sato T Kubota M . Association of difference between eGFR from cystatin C and creatinine and serum GDF-15 with adverse outcomes in diabetes mellitus. J Cachexia Sarcopenia Muscle. (2025) 16:e70011. doi: 10.1002/jcsm.70011 40700030 PMC12285683

[31] Delanaye P Flamant M Vidal-Petiot E Bjork J Nyman U Grubb A . Discordant results between creatinine- and cystatin C-based equations for estimating GFR. Kidney Int Rep. (2025) 10:1248–59. doi: 10.1016/j.ekir.2025.01.030 40303200 PMC12034923

[32] Wang Y Adingwupu OM Shlipak MG Doria A Estrella MM Froissart M . Discordance between creatinine-based and cystatin C-based estimated GFR: Interpretation according to performance compared to measured GFR. Kidney Med. (2023) 5:100710. doi: 10.1016/j.xkme.2023.100710 37753251 PMC10518599

[33] McCoy IE Yang J Go AS Rincon-Choles H Taliercio J Rosas SE . Complex etiologies of the discordance between cystatin C- and creatinine-based estimated GFR and its adverse associations: Findings from the CRIC study. Am J Kidney Dis. (2025) 86:192–201. doi: 10.1053/j.ajkd.2025.03.018 40373998 PMC12818706

[34] Hernandez BN Wieruszewski PM Barreto JN Cole KC Damani S Kane-Gill SL . Challenges in renally eliminated medication use: Evaluating cystatin C and serum creatinine eGFR discordance. Pharmacotherapy. (2024) 44:898–906. doi: 10.1002/phar.4627 39601345 PMC11693467

[35] Williams V Parbhoo R Doepker B Murphy C Wardlow L Kissling K . Implementing use of cystatin C-guided estimated glomerular filtration rate for medication dosing at an academic medical center. J Am Coll Clin Pharm. (2025) 8:376–84. doi: 10.1002/jac5.7003542587375

[36] Rule AD Glassock RJ . Cystatin C and the misdiagnosis of CKD in older adults. Nat Rev Nephrol. (2024) 20:560–1. doi: 10.1038/s41581-024-00852-y 38831050

[37] Xiong K Zhang S Zhong P Zhu Z Chen Y Huang W . Serum cystatin C for risk stratification of prediabetes and diabetes populations. Diabetes Metab Syndr. (2023) 17:102882. doi: 10.1016/j.dsx.2023.102882 37898064

[38] Chen DC Lees JS Lu K Scherzer R Rutherford E Mark PB . Differential associations of cystatin C versus creatinine-based kidney function with risks of cardiovascular event and mortality among South Asian individuals in the UK Biobank. J Am Heart Assoc. (2023) 12:e027079. doi: 10.1161/JAHA.122.027079 36695320 PMC9973614

[39] Cavalier E Zima T Datta P Makris K Schaeffner E Langlois M . Recommendations for European laboratories based on the KDIGO 2024 clinical practice guideline for the evaluation and management of chronic kidney disease. Clin Chem Lab Med. (2025) 63:525–34. doi: 10.1515/cclm-2024-1082 39584585

[40] Sacks DB Arnold M Bakris GL Bruns DE Horvath AR Lernmark A . Guidelines and recommendations for laboratory analysis in the diagnosis and management of diabetes mellitus. Clin Chem. (2023) 69:808–68. doi: 10.1093/clinchem/hvad080 37473453 PMC12376302

