# Supplementary material for: Targeted cystatin C testing for large negative eGFR discordance in adults with diabetes: development and external validation of a machine-learning model
Source: Front Endocrinol (Lausanne). 2026 Oct 29;17:2002540. doi: 10.3389/fendo.2026.2002540 (PMC13754737; doi:10.3389/fendo.2026.2002540)
Supplement: Supplementary file 2 [file Table1.docx]

Supplementary Material

# Contents

Supplementary Methods 1–4; Tables S1–S12; Figures S1–S5.

# Supplementary Methods 1

## Cohort construction, index measurements, and phenotype definitions

Adults aged ≥18 years with a recorded clinical diagnosis of diabetes were included without requiring reliable separation of type 1 and type 2 diabetes. The development source was Wanbei Hospital from May 1, 2022, through November 30, 2023. The independent external source comprised available February and April–December 2024 records from the First Affiliated Hospital of Anhui Medical University. January and March records were inadvertently omitted during retrospective data compilation, an administrative omission unrelated to patient outcomes or model performance; the external dataset therefore did not provide continuous coverage of the full year. Each participant contributed the earliest eligible index observation within the permitted period. Same-date ties were ordered by specimen identifier. Reliable patient identifiers were required, with linkage permitted only when a unique identity could be established within the study period.

Serum creatinine and cystatin C were measured from the same blood specimen using Atellica CH 930 analyzers (Siemens Healthineers) at both hospitals. Serum creatinine was measured using the sarcosine oxidase enzymatic method with reagents manufactured by Anhui Tongzhi Biological Engineering Co., Ltd., with calibration traceable to GBW(E)091004 and the isotope dilution mass spectrometry (IDMS) reference system. Serum cystatin C was measured using a latex-enhanced immunoturbidimetric assay with reagents manufactured by Anhui Iprocom Biotechnology Co., Ltd., with calibration traceable to ERM-DA471/IFCC. Both hospitals used the same analytical instrument model, reagent products, and measurement methods, without changes during their respective study periods. Chemistry and lipid measurements came from the index specimen. CBC and glycated hemoglobin (HbA1c) panels were selected as the nearest eligible panels from 7 days before the index date through the index date, inclusive, without crossing the cohort-period boundary. Components from different panels were not combined. The rule did not require complete extended-CBC or lipid panels. Exact within-day result-release order was unavailable.

After review of patient-level and laboratory-module missingness, modeling eligibility required valid measured white blood cell count (WBC), hemoglobin (HGB), and platelet count (PLT). Missing measurements were not imputed to bypass eligibility, and no additional lipid or extended-CBC completeness requirement was imposed. The phenotype-eligible populations contained 2,012 development and 1,775 external participants. After the completeness rule and sentinel-value quality control, the final modeling cohorts contained 1,170 participants with 214 D2 events and 1,619 participants with 112 D2 events, respectively. One development record with an invalid all-zero CBC had no valid replacement within the prespecified window and was excluded; a later out-of-window CBC was not substituted.

Creatinine was expressed in mg/dL (µmol/L divided by 88.4) and cystatin C in mg/L. eGFRcr used the CKD-EPI 2021 creatinine equation [1]:

eGFRcr = 142 × min(SCr/κ, 1)^α^ × max(SCr/κ, 1)^−1.200^ × 0.9938^Age^ × 1.012 (if female).

κ is 0.7 for females and 0.9 for males; α is −0.241 and −0.302, respectively. eGFRcys used the CKD-EPI 2012 cystatin C equation [2]:

eGFRcys = 133 × min(Cys-C/0.8, 1)^−0.499^ × max(Cys-C/0.8, 1)^−1.328^ × 0.996^Age^ × 0.932 (if female).

D2 was defined as eGFRcys/eGFRcr <0.70; the strict inequality corresponds to eGFRcys being more than 30% lower than eGFRcr. D1 was eGFRcys − eGFRcr ≤−15 mL/min/1.73 m². D3 was eGFRcys <60 mL/min/1.73 m² among participants with eGFRcr ≥60 mL/min/1.73 m², and prevalence used only this eligible subset as its denominator. These single-observation renal phenotypes did not establish chronicity or independently adjudicated kidney disease.

# Supplementary Methods 2

## Variable processing, model development, and interpretation

The initial universe contained 49 demographic and clinical laboratory candidates. Availability was recalculated after formation of the modeling cohorts. Variables with >25% missingness in either primary cohort were excluded (49 to 41); creatinine was removed for structural redundancy with mandatory eGFRcr (41 to 40); and CO₂ was excluded because cross-center measurement equivalence was uncertain (40 to 39). This feasibility review used cross-center availability and harmonization information, not external model performance. Cystatin C and its derived phenotype measurements were not candidate predictors. Harmonization used analyte identity and consistent units. A/G was uniformly calculated as albumin/globulin and was missing if either component was missing or globulin was nonpositive. Source-reported non-HDL cholesterol was not universally reconstructed. Lipid and HbA1c exclusions reflected availability rather than model performance.

Pairwise-complete Spearman correlations among the 39 candidates were reviewed without imputation. Clinical redundancy review retained HGB rather than red blood cell count/hematocrit; mean corpuscular volume and mean corpuscular hemoglobin concentration rather than mean corpuscular hemoglobin; differential absolute leukocyte counts rather than WBC and the corresponding percentages; and albumin plus A/G rather than total protein/globulin. This yielded the fixed 28-variable Full pool. Table S1 reports available measurements and missing-data percentages for all 49 candidate variables in each cohort. Figure S1 shows the pre-redundancy correlation stage, not the final Full pool.

Seven algorithms were evaluated: logistic regression, support vector machine, k-nearest neighbors, decision tree, random forest, XGBoost, and LightGBM. Median imputation was fitted in training folds only. Standardization was also fitted within training folds for logistic regression, support vector machine, and k-nearest neighbors. Sex was coded as 0 for female and 1 for male. No class weighting, oversampling, or synthetic resampling was used. Hyperparameters were evaluated with five stratified folds, shuffling enabled, and random state 42; grids and selected settings are reported in Table S3.

Average precision, reported as AUPRC, was the primary selection metric. Tuning and subsequent pooled patient-level OOF evaluation used the same five-fold partition. Development results therefore represent model-selection performance, not nested or independent internal validation. Preprocessing remained confined to training folds; confidence intervals conditional on saved predictions do not remove selection optimism.

The two leading Full-pool algorithms, XGBoost and random forest, underwent SHAP-ranked feature reduction from 28 to 4 predictors while always retaining age, sex, and eGFRcr. Hyperparameters remained unchanged. Within each algorithm, the highest-AUPRC row was the reference, choosing the larger panel for an exact tie. A panel qualified as near-optimal if AUROC and AUPRC were each no more than 0.01 below the reference and Brier score no more than 0.01 above it. The smallest qualifying panel was provisional. Between algorithms, an absolute AUPRC difference ≥0.01 determined preference; otherwise AUROC and Brier were compared, followed by parsimony when differences remained within the tolerances. Global SHAP ranking was generated using the full development cohort before the reduced-panel cross-validation; this introduces feature-selection reuse into development estimates.

In an added development-stage stability check, not an analysis prespecified at study initiation, the neighboring eight- and nine-predictor XGBoost panels were compared using ten repeats of stratified five-fold cross-validation (seeds 42–51), with identical folds for both panels and fixed hyperparameters. Metrics in each repeat used pooled held-out predictions. Small but directionally consistent gains supported retention of alkaline phosphatase (ALP; Table S4). The final predictors were age, sex, eGFRcr, HGB, PLT, blood urea nitrogen (BUN), albumin (ALB), ALP, and glucose (GLU). Main development estimates used the original seed-42 pooled OOF predictions, not the repeated-cross-validation mean. The clinical core was logistic regression with age, sex, and eGFRcr (L2 penalty, C=1, liblinear solver). No final diagnostic probability cutoff was introduced. The documented feature-selection and model-fitting procedures did not use external outcomes to select predictors or optimize hyperparameters.

Final-model interpretation used mean absolute SHAP values, beeswarm summaries, and predictor dependence plots. XGBoost SHAP values were reported on the raw-margin/log-odds scale. SHAP ranking and dependence patterns were treated as descriptive model behavior rather than causal effects or clinical thresholds. Sensitivity, specificity, and F1 score displayed in main Figure 2E were descriptive threshold-dependent development metrics and did not contribute to the prespecified AUPRC/AUROC/Brier-based feature-selection rule.

# Supplementary Methods 3

## **External validation, targeted testing, and statistical analysis**

The final nine-variable XGBoost pipeline and the three-variable clinical core were fitted using the complete development cohort and applied unchanged to the independent external validation cohort. Primary AUROC, AUPRC, Brier score, and decision-curve analyses used the original external probabilities. Patient-level percentile 95% confidence intervals were estimated from 2,000 bootstrap resamples within each cohort, with identical resamples used for paired XGBoost-versus-core differences. Development confidence intervals were conditional on the saved pooled out-of-fold predictions and did not repeat model or feature selection.

Calibration intercept and slope were estimated jointly by logistic regression of D2 status on the logit of predicted probability; calibration-in-the-large (CITL) was estimated with the slope fixed at 1. Original external calibration metrics and the raw calibration curve were retained in Table S6A and Figure S3, with raw calibration also displayed in main Figure 3D. Because the original probabilities showed substantial center-level calibration drift, post-hoc local logistic recalibration was additionally assessed using five stratified folds. Recalibration parameters estimated in four folds were applied only to the held-out fold, so that each participant received one cross-fitted locally recalibrated probability. This procedure did not retrain XGBoost and was not treated as additional independent validation. The local cross-fitted calibration figure is provided as Figure S4.

Decision-curve net benefit was calculated as TP/N − FP/N × pt/(1−pt), with test-all and test-none strategies as references. Original external probabilities remain primary. The 0–0.12 interval was a historical display range, without an established project-specific clinical decision-threshold basis. Figure S5 additionally displays 0–0.50 as a descriptive sensitivity range, including the cross-fitted local recalibration results. Decision-curve analysis is exploratory; a display interval is not an optimized or validated clinical cutoff.

For targeted cystatin C testing, participants were ranked by pooled development out-of-fold probabilities or original external probabilities. Fixed testing allocations of 10%, 20%, 30%, 40%, and 50% were evaluated. The number selected at each allocation was floor(N x allocation), and tied probabilities at an allocation boundary were resolved reproducibly without reference to D2 status. Case capture was defined as detected D2 cases divided by all D2 cases; testing yield as detected D2 cases divided by the number selected; tests per D2 detected as the number selected divided by detected cases; and enrichment as testing yield divided by cohort D2 prevalence. Each of 2,000 patient-level bootstrap resamples was reranked before application of the fixed testing allocations.

The identical XGBoost top-20% selection was used for four-group phenotype characterization: D2-negative/model-negative, D2-negative/model-positive, D2-positive/model-negative, and D2-positive/model-positive. The prespecified primary contrast compared the two D2-negative groups. Binary outcomes were summarized primarily using absolute risk differences with Newcombe 95% confidence intervals [3], with risk ratios reported secondarily; Wilson intervals were used for group proportions [4]. Continuous outcomes were compared using differences in medians with percentile bootstrap intervals. These descriptive comparisons were not adjusted for multiplicity. Related cystatin C-based phenotypes were not considered independent confirmation of kidney disease.

Baseline continuous variables were summarized as median [Q1, Q3] and compared using two-sided Mann-Whitney U tests. Categorical variables were summarized as n (%) and compared using chi-square or Fisher exact tests, as appropriate. Descriptive analyses used available observations without imputation. Additional model comparisons and sensitivity analyses are detailed in Supplementary Methods 4.

# Supplementary Methods 4

## Additional comparisons and sensitivity analyses

Additional comparisons and sensitivity analyses retained the final nine-variable XGBoost, the D2 definition, and cohort membership. Two unpenalized logistic regressions used eGFRcr alone and eGFRcr, BUN and ALB, respectively. Median imputation and standardization were fitted only in each training fold, using the saved five-fold development assignments; full-development pipelines were applied externally. The original clinical core retained its historical specification. A separate hand-calculable continuous score rounded the three-variable model coefficients to three significant digits, including fold-specific rounding for development evaluation. No clinical thresholds, interactions or nonlinear terms were optimized. Equations are in Table S5C.

Original-probability model performance and paired differences are reported in Table S5A–B. Table S5D gives descriptive operating points at achieved sensitivity of at least 80% and 90% on each cohort’s own empirical ROC curve; these are not development cutoffs transported externally, and specificity can still depend on case mix. Average precision depends on prevalence; the observed AP change cannot be assigned entirely to prevalence rather than changes in discrimination or case mix.

For each testing allocation, every strategy scored the same study cohort under the same testing budget; rankings and selected individuals could differ between strategies. Ties were resolved using the first 64 bits of SHA256(42|cohort|study_id), without consulting outcome status. Each of 2,000 paired patient bootstrap resamples was reranked for each strategy. Repeated sampled copies used sampling position as a final tie breaker. Percentile intervals used the 2.5th and 97.5th percentiles. No model was refitted in these bootstrap analyses, and development intervals do not account for model-selection uncertainty. Table S7C reports paired recall increments across all five budgets; all model-specific utility intervals are in S7A–B.

Local recalibration used the previously defined five external folds, estimating intercept and slope on the other four folds. It is an external-cohort local-adaptation sensitivity analysis, not another independent external validation. Different fold-specific transformations can change pooled rankings. Pointwise DCA intervals in Table S6C condition on saved probabilities and do not refit the recalibration model in bootstrap resamples; they do not capture all recalibration-parameter uncertainty and are not simultaneous confidence bands.

Included and excluded participants were compared within the original phenotype-eligible populations. Descriptive copies treated the invalid all-zero development CBC module as missing. No excluded data were imputed to meet eligibility. Standardized mean differences (SMDs) used the difference in means divided by the square root of the average sample variances for continuous variables, and pooled Bernoulli variance for binary variables. Medians, quartiles and valid/missing counts accompany SMDs. Between-center comparisons and testing coverage use only the available extraction files and cannot establish complete coverage of all adults with diabetes at either hospital.

Complete-case sensitivity retained the original development OOF and external scores without refitting. A mean-imputation sensitivity analysis retained the fixed XGBoost structure and original folds, replacing only median with mean imputation; it does not replace the original model. The frozen D2 scores were also evaluated against D1 (eGFRcys−eGFRcr ≤−15 mL/min/1.73 m²) without training a D1 model. These related phenotypes do not independently verify kidney disease.

Repeated SCr/CysC pairs were linked using the original reliable-patient identity rules and verified against original LIS values, units and specimen identifiers. The primary window was 30–365 days and the sensitivity window 7–365 days; the earliest valid repeat was selected, with specimen identifiers resolving same-date ties. Follow-up remained within the original extraction period and is subject to right censoring. Agreement intervals used Wilson’s method; kappa intervals used 2,000 patient bootstrap resamples. Repeated-measurement availability was selective, and no measured GFR reference was available. Repeat stability is therefore not validation of GFR accuracy or proof of persistent CKD.

All added model comparisons, alternative-phenotype assessments and repeat-subset analyses are exploratory and were not adjusted for multiple comparisons. The crude ratios of D2 events to the nine input variables were 214/9=23.78 in development and 112/9=12.44 externally; these are not XGBoost effective degrees of freedom, formal sample-size calculations or evidence of adequate statistical power. Additional statistical analyses were conducted in Python 3.13; versioned package and source records are retained with the analysis.

The available records did not reliably capture indications for ordering cystatin C at either hospital, limiting assessment of testing selection mechanisms. Reliable type 1/type 2 diabetes classification was unavailable; therefore, diabetes-type subgroup performance analyses were not undertaken.

## Supplementary references

[1] Inker LA, Eneanya ND, Coresh J, Tighiouart H, Wang D, Sang Y, et al. New Creatinine- and Cystatin C-Based Equations to Estimate GFR without Race. N Engl J Med (2021) 385:1737–1749. doi:10.1056/NEJMoa2102953.

[2] Inker LA, Schmid CH, Tighiouart H, Eckfeldt JH, Feldman HI, Greene T, et al. Estimating glomerular filtration rate from serum creatinine and cystatin C. N Engl J Med (2012) 367:20–29. doi:10.1056/NEJMoa1114248.

[3] Newcombe RG. Interval estimation for the difference between independent proportions: comparison of eleven methods. Stat Med (1998) 17:873–890. doi:10.1002/(SICI)1097-0258(19980430)17:8<873::AID-SIM779>3.0.CO;2-I.

[4] Wilson EB. Probable inference, the law of succession, and statistical inference. J Am Stat Assoc (1927) 22:209–212. doi:10.1080/01621459.1927.10502953.

## Table S1 Candidate-variable availability and missingness

| **Variables** | **Development available n** | **Missing data (%)** | **External validation available n** | **Missing data (%)** |
| --- | --- | --- | --- | --- |
| Demographics and renal function |  |  |  |  |
| Age, years | 1170 | 0.00 | 1619 | 0.00 |
| Sex | 1170 | 0.00 | 1619 | 0.00 |
| SCr, mg/dL | 1170 | 0.00 | 1619 | 0.00 |
| eGFRcr, mL/min/1.73 m² | 1170 | 0.00 | 1619 | 0.00 |
| Hematology |  |  |  |  |
| WBC, 10⁹/L | 1170 | 0.00 | 1619 | 0.00 |
| RBC, 10¹²/L | 1170 | 0.00 | 1619 | 0.00 |
| HGB, g/L | 1170 | 0.00 | 1619 | 0.00 |
| HCT, % | 1170 | 0.00 | 1619 | 0.00 |
| MCV, fL | 1170 | 0.00 | 1619 | 0.00 |
| MCH, pg | 1170 | 0.00 | 1619 | 0.00 |
| MCHC, g/L | 1170 | 0.00 | 1619 | 0.00 |
| RDW, % | 1170 | 0.00 | 1618 | 0.06 |
| PLT, 10⁹/L | 1170 | 0.00 | 1619 | 0.00 |
| NEUT#, 10⁹/L | 1170 | 0.00 | 1619 | 0.00 |
| LYMPH#, 10⁹/L | 1170 | 0.00 | 1619 | 0.00 |
| MONO#, 10⁹/L | 1170 | 0.00 | 1619 | 0.00 |
| EO#, 10⁹/L | 1170 | 0.00 | 1619 | 0.00 |
| BASO#, 10⁹/L | 1170 | 0.00 | 1619 | 0.00 |
| NEUT%, % | 1170 | 0.00 | 1619 | 0.00 |
| LYMPH%, % | 1170 | 0.00 | 1619 | 0.00 |
| MONO%, % | 1170 | 0.00 | 1619 | 0.00 |
| EO%, % | 1170 | 0.00 | 1619 | 0.00 |
| BASO%, % | 1170 | 0.00 | 1619 | 0.00 |
| MPV, fL | 1164 | 0.51 | 1590 | 1.79 |
| Biochemistry |  |  |  |  |
| BUN, mmol/L | 1170 | 0.00 | 1619 | 0.00 |
| UA, µmol/L | 1170 | 0.00 | 1619 | 0.00 |
| ALB, g/L | 1150 | 1.71 | 1552 | 4.14 |

## Table S1 Candidate-variable availability and missingness continued

| **Variables** | **Development available n** | **Missing data (%)** | **External validation available n** | **Missing data (%)** |
| --- | --- | --- | --- | --- |
| TP, g/L | 1150 | 1.71 | 1552 | 4.14 |
| GLB, g/L | 1150 | 1.71 | 1552 | 4.14 |
| A/G | 1150 | 1.71 | 1552 | 4.14 |
| ALT, U/L | 1150 | 1.71 | 1552 | 4.14 |
| AST, U/L | 1151 | 1.62 | 1552 | 4.14 |
| ALP, U/L | 1150 | 1.71 | 1552 | 4.14 |
| GGT, U/L | 1150 | 1.71 | 1552 | 4.14 |
| TBIL, µmol/L | 1150 | 1.71 | 1552 | 4.14 |
| Na, mmol/L | 1156 | 1.20 | 1570 | 3.03 |
| K, mmol/L | 1155 | 1.28 | 1570 | 3.03 |
| Cl, mmol/L | 1156 | 1.20 | 1570 | 3.03 |
| Ca, mmol/L | 1158 | 1.03 | 1565 | 3.34 |
| GLU, mmol/L | 1136 | 2.91 | 1520 | 6.11 |
| LDH, U/L | 481 | 58.89 | 1532 | 5.37 |
| P, mmol/L | 0 | 100.00 | 1565 | 3.34 |
| CO₂, mmol/L | 1158 | 1.03 | 1565 | 3.34 |
| HbA1c, % | 972 | 16.92 | 0 | 100.00 |
| TC, mmol/L | 1143 | 2.31 | 934 | 42.31 |
| HDL-C, mmol/L | 1146 | 2.05 | 934 | 42.31 |
| LDL-C, mmol/L | 1146 | 2.05 | 934 | 42.31 |
| TG, mmol/L | 1146 | 2.05 | 934 | 42.31 |
| Non-HDL-C, mmol/L | 0 | 100.00 | 934 | 42.31 |

Values are the numbers of participants with available measurements and the corresponding percentages of missing data in the final modeling cohorts (development, n = 1,170; external validation, n = 1,619). Abbreviations: SCr, serum creatinine; eGFRcr, creatinine-based estimated glomerular filtration rate; WBC/RBC, white/red blood cell count; HGB, hemoglobin; HCT, hematocrit; MCV/MCH/MCHC, mean corpuscular volume/hemoglobin/hemoglobin concentration; RDW, red-cell distribution width; PLT, platelet count; MPV, mean platelet volume; NEUT/LYMPH/MONO/EO/BASO, neutrophils/lymphocytes/monocytes/eosinophils/basophils (#, absolute count; %, percentage); BUN, blood urea nitrogen; UA, uric acid; ALB, albumin; TP, total protein; GLB, globulin; A/G, albumin-to-globulin ratio; ALT/AST, alanine/aspartate aminotransferase; ALP, alkaline phosphatase; GGT, gamma-glutamyl transferase; TBIL, total bilirubin; Na/K/Cl/Ca/P, sodium/potassium/chloride/calcium/phosphorus; GLU, glucose; LDH, lactate dehydrogenase; CO₂, carbon dioxide; HbA1c, glycated hemoglobin; TC, total cholesterol; HDL-C/LDL-C, high-/low-density lipoprotein cholesterol; TG, triglycerides.

## Table S2 External baseline characteristics

| **Characteristic** | **Unit** | **D2-negative (n = 1507)** | **D2-positive (n = 112)** | **P value** |
| --- | --- | --- | --- | --- |
| Demographics / clinical core |  |  |  |  |
| age | years | 61.0 [52.0, 70.0] | 71.0 [59.0, 79.0] | < 0.001 |
| Sex (male), n (%) | — | 985 (65.4%) | 72 (64.3%) | 0.817 |
| Hematology |  |  |  |  |
| HGB | g/L | 133.00 [118.00, 146.00] | 105.50 [92.50, 118.25] | < 0.001 |
| MCV | fL | 91.20 [88.00, 94.10] | 92.45 [88.15, 95.45] | 0.118 |
| MCHC | g/L | 330.00 [322.00, 338.00] | 322.00 [314.75, 332.25] | < 0.001 |
| RDW | % | 12.90 [12.40, 13.60] | 14.60 [13.20, 16.23] | < 0.001 |
| PLT | 10⁹/L | 205.00 [166.00, 254.50] | 177.00 [126.25, 246.75] | < 0.001 |
| MPV | fL | 10.80 [10.10, 11.60] | 11.00 [10.30, 11.85] | 0.079 |
| NEUT# | 10⁹/L | 3.72 [2.94, 5.01] | 4.54 [3.00, 7.06] | 0.002 |
| LYMPH# | 10⁹/L | 1.72 [1.33, 2.17] | 1.27 [0.95, 1.85] | < 0.001 |
| MONO# | 10⁹/L | 0.41 [0.32, 0.52] | 0.45 [0.34, 0.60] | 0.014 |
| EO# | 10⁹/L | 0.12 [0.07, 0.20] | 0.08 [0.04, 0.20] | 0.003 |
| BASO# | 10⁹/L | 0.03 [0.02, 0.05] | 0.03 [0.01, 0.04] | < 0.001 |
| Biochemistry |  |  |  |  |
| BUN | mmol/L | 5.74 [4.55, 7.38] | 6.50 [4.93, 8.87] | 0.007 |
| UA | µmol/L | 310.00 [249.00, 380.00] | 306.00 [206.25, 384.50] | 0.262 |
| ALB | g/L | 41.50 [38.10, 44.30] | 35.10 [30.50, 39.12] | < 0.001 |
| A/G | ratio | 1.73 [1.47, 1.96] | 1.41 [1.05, 1.68] | < 0.001 |
| ALT | U/L | 19.00 [14.00, 27.00] | 19.00 [13.00, 27.25] | 0.720 |
| AST | U/L | 17.00 [14.00, 23.00] | 20.00 [14.00, 35.25] | 0.007 |

## Table S2 External baseline characteristics continued

| **Characteristic** | **Unit** | **D2-negative (n = 1507)** | **D2-positive (n = 112)** | **P value** |
| --- | --- | --- | --- | --- |
| ALP | U/L | 76.00 [62.00, 95.00] | 85.50 [65.00, 123.50] | < 0.001 |
| GGT | U/L | 21.00 [14.00, 34.00] | 30.50 [16.00, 49.50] | < 0.001 |
| TBIL | µmol/L | 13.00 [10.20, 16.40] | 11.50 [9.05, 15.90] | 0.063 |
| Na | mmol/L | 139.30 [137.50, 140.70] | 139.20 [136.00, 141.40] | 0.800 |
| K | mmol/L | 4.02 [3.77, 4.31] | 3.98 [3.51, 4.41] | 0.202 |
| Cl | mmol/L | 100.00 [98.10, 102.00] | 101.50 [98.50, 104.70] | < 0.001 |
| Ca | mmol/L | 2.21 [2.13, 2.28] | 2.15 [2.02, 2.24] | < 0.001 |
| GLU | mmol/L | 7.53 [6.00, 9.90] | 7.51 [5.79, 9.36] | 0.382 |
| Renal core |  |  |  |  |
| eGFRcr_2021 | mL/min/1.73 m² | 98.70 [80.55, 108.50] | 91.15 [69.43, 99.55] | < 0.001 |
| Phenotype characterization |  |  |  |  |
| SCr_mg_dL | mg/dL | 0.77 [0.62, 0.98] | 0.84 [0.64, 1.01] | 0.303 |
| CysC_mg_L | mg/L | 0.80 [0.68, 0.99] | 1.33 [1.12, 1.63] | < 0.001 |
| eGFRcys_2012 | mL/min/1.73 m² | 98.88 [74.65, 112.78] | 51.16 [37.36, 61.81] | < 0.001 |
| eGFR_difference | mL/min/1.73 m² | 1.83 [-5.94, 8.10] | -32.21 [-42.68, -27.46] | < 0.001 |
| eGFR_ratio | ratio | 1.02 [0.93, 1.09] | 0.61 [0.52, 0.66] | < 0.001 |

Continuous variables are median [Q1, Q3]; categorical variables are n (%). Two-sided Mann–Whitney U tests were used for continuous variables; chi-square or Fisher exact tests, as appropriate, for categorical variables. Available values were used without descriptive imputation. Variable order, units and statistical format match the development baseline table. Cystatin C and cystatin C-derived measurements were used for phenotype definition/characterization and were not included in the machine-learning predictor set. Sex was coded as 0 = female and 1 = male.

## Table S3A Development performance of seven Full-pool algorithms

| **Model** | **Predictors** | **AUROC** | **AUPRC** | **Brier** |
| --- | --- | --- | --- | --- |
| XGBoost | 28 | 0.854 | 0.584 | 0.106 |
| Random Forest | 28 | 0.850 | 0.574 | 0.107 |
| LightGBM | 28 | 0.854 | 0.565 | 0.108 |
| Support Vector Machine | 28 | 0.833 | 0.541 | 0.112 |
| K-nearest Neighbors | 28 | 0.831 | 0.504 | 0.123 |
| Logistic Regression | 28 | 0.814 | 0.500 | 0.118 |
| Decision Tree | 28 | 0.779 | 0.448 | 0.128 |

Development: n = 1,170; D2-positive n = 214. Metrics are pooled patient-level OOF estimates from the 28-variable Full pool. AUPRC is average precision; lower Brier is better. Tuning and OOF evaluation used the same five-fold partition, so these are development/model-selection estimates rather than independent internal-validation estimates. The 28-variable logistic-regression model shown here was one of the seven candidate algorithms and is distinct from the separately specified three-variable clinical core comparator (age, sex, and eGFRcr).

## Table S3B Hyperparameter grids and selected settings

| **Algorithm** | **Search grid** | **Selected settings** |
| --- | --- | --- |
| Logistic Regression | C: 0.01, 0.1, 1, 10, 100; penalty: L1, L2 | solver = liblinear; penalty = l1; C = 1; max_iter = 10000 |
| Support Vector Machine | C: 0.1, 1, 10, 100; gamma: scale, 0.01, 0.1; RBF kernel | kernel = rbf; probability = True; C = 1; gamma = scale |
| K-nearest Neighbors | k: 5, 15, 25, 45; weights: uniform, distance; p: 1, 2 | n_neighbors = 45; p = 1; weights = distance |
| Decision Tree | max_depth: 3, 5, 8; min_samples_leaf: 5, 10, 20; criterion: gini, entropy | min_samples_split = 2; criterion = entropy; max_depth = 5; min_samples_leaf = 10 |
| Random Forest | max_depth: 5, 10, unlimited; min_samples_leaf: 2, 5; max_features: sqrt, 0.7; 300 trees | n_estimators = 300; min_samples_split = 2; max_depth = 5; max_features = sqrt; min_samples_leaf = 5 |
| XGBoost | n_estimators: 150, 300; learning_rate: 0.03, 0.1; max_depth: 2, 3; min_child_weight: 1, 5 | scale_pos_weight = 1; eval_metric = logloss; objective = binary: logistic; tree_method = hist; subsample = 0.8; colsample_bytree = 0.8; reg_lambda = 1; learning_rate = 0.03; max_depth = 2; min_child_weight = 1; n_estimators = 300 |
| LightGBM | n_estimators: 150, 300; learning_rate: 0.03, 0.1; num_leaves: 7, 15; min_child_samples: 20, 40 | is_unbalance = False; scale_pos_weight = 1; objective = binary; subsample = 0.8; subsample_freq = 1; colsample_bytree = 0.8; max_depth = -1; learning_rate = 0.03; min_child_samples = 20; n_estimators = 150; num_leaves = 15 |

Search sizes were 10, 12, 16, 18, 12, 16 and 16 combinations, respectively. All used identical five stratified folds and average precision for selection. Median imputation was fold-specific; scaling applied to logistic regression, support vector machine and k-nearest neighbors. Class weights were unset and scale_pos_weight = 1; no oversampling was used.

## Table S4A Feature-count reduction

| **k** | **XGBoost AUROC** | **XGBoost AUPRC** | **XGBoost Brier** | **Near- optimal** | **Random forest AUROC** | **Random forest AUPRC** | **Random forest Brier** | **Near- optimal** |
| --- | --- | --- | --- | --- | --- | --- | --- | --- |
| 4 | 0.824737 | 0.493007 | 0.115885 | No | 0.833511 | 0.506614 | 0.113258 | No |
| 5 | 0.840398 | 0.533692 | 0.109860 | No | 0.851435 | 0.543462 | 0.107410 | No |
| 6 | 0.854163 | 0.561672 | 0.105025 | No | 0.855605 | 0.540863 | 0.106368 | No |
| 7 | 0.862834 | 0.588297 | 0.102196 | No | 0.852550 | 0.549747 | 0.106258 | No |
| 8 | 0.862501 | 0.595517 | 0.101350 | Yes | 0.857472 | 0.568368 | 0.104520 | No |
| 9 | 0.863308 | 0.600190 | 0.102354 | Yes | 0.853674 | 0.570566 | 0.104071 | No |
| 10 | 0.864325 | 0.602469 | 0.102028 | Yes | 0.850829 | 0.570210 | 0.104796 | No |
| 11 | 0.862961 | 0.583031 | 0.104447 | No | 0.850756 | 0.574560 | 0.104984 | Yes |
| 12 | 0.860155 | 0.585311 | 0.104836 | No | 0.849602 | 0.569342 | 0.105308 | No |
| 13 | 0.859618 | 0.588412 | 0.104386 | No | 0.850707 | 0.568947 | 0.105436 | No |
| 14 | 0.860644 | 0.602529 | 0.103591 | Yes | 0.849465 | 0.562965 | 0.106607 | No |
| 15 | 0.860253 | 0.596034 | 0.103623 | Yes | 0.849900 | 0.558889 | 0.106428 | No |
| 16 | 0.859569 | 0.602051 | 0.104140 | Yes | 0.851787 | 0.583738 | 0.105196 | Yes |
| 17 | 0.857271 | 0.598093 | 0.103825 | Yes | 0.851244 | 0.582540 | 0.105819 | Yes |
| 18 | 0.855697 | 0.594128 | 0.104006 | Yes | 0.849333 | 0.576316 | 0.105893 | Yes |
| 19 | 0.855893 | 0.592859 | 0.105215 | Yes | 0.849695 | 0.567543 | 0.106126 | No |
| 20 | 0.855898 | 0.595603 | 0.105048 | Yes | 0.851758 | 0.573358 | 0.106064 | No |
| 21 | 0.856206 | 0.595744 | 0.104926 | Yes | 0.852227 | 0.571392 | 0.106595 | No |
| 22 | 0.854803 | 0.593689 | 0.105214 | Yes | 0.851601 | 0.572125 | 0.106388 | No |
| 23 | 0.853209 | 0.582368 | 0.105707 | No | 0.850150 | 0.564433 | 0.107079 | No |
| 24 | 0.853811 | 0.586645 | 0.105590 | No | 0.848928 | 0.570009 | 0.107041 | No |
| 25 | 0.851782 | 0.576698 | 0.106491 | No | 0.849842 | 0.571717 | 0.106746 | No |
| 26 | 0.850648 | 0.574336 | 0.107289 | No | 0.852027 | 0.564837 | 0.106568 | No |
| 27 | 0.852789 | 0.582396 | 0.106138 | No | 0.850878 | 0.569023 | 0.106559 | No |
| 28 | 0.854036 | 0.584300 | 0.105672 | No | 0.850306 | 0.574486 | 0.106656 | Yes |

k, number of predictors. The reference configurations with the highest pooled OOF AUPRC were k = 14 for XGBoost and k = 16 for random forest. Near-optimal panels required AUROC and AUPRC decreases ≤0.01 and a Brier increase ≤0.01 relative to the corresponding reference configuration. Age, sex, and eGFRcr were always retained. The smallest qualifying panels in the single five-fold analysis contained eight predictors for XGBoost and 11 for random forest. The final nine-predictor XGBoost panel was retained after the neighboring-panel stability comparison below, without selection based on external performance.

## Table S4B Added eight-versus-nine stability comparison

| **Panel / contrast** | **Metric** | **Mean ± SD** | **Median [Q1, Q3]** | **Range** |
| --- | --- | --- | --- | --- |
| 8 predictors | AUROC | 0.860734 ± 0.003152 | 0.861350 [0.860907, 0.862339] | 0.854637 to 0.864980 |
| 8 predictors | AUPRC | 0.601236 ± 0.007326 | 0.601132 [0.598121, 0.605844] | 0.585968 to 0.611061 |
| 8 predictors | Brier | 0.101362 ± 0.000986 | 0.101142 [0.100821, 0.101570] | 0.100265 to 0.103646 |
| 9 predictors | AUROC | 0.864254 ± 0.002957 | 0.863992 [0.862273, 0.866233] | 0.859662 to 0.868636 |
| 9 predictors | AUPRC | 0.607487 ± 0.008365 | 0.604952 [0.601277, 0.609722] | 0.598579 to 0.623311 |
| 9 predictors | Brier | 0.100939 ± 0.001344 | 0.100855 [0.099947, 0.101921] | 0.098892 to 0.103077 |
| 9 minus 8 | AUROC | 0.003520 ± 0.002559 | 0.003512 [0.001012, 0.005432] | 0.000499 to 0.007444 |
| 9 minus 8 | AUPRC | 0.006251 ± 0.004878 | 0.005636 [0.004757, 0.009909] | -0.003048 to 0.012611 |
| 9 minus 8 | Brier | -0.000423 ± 0.000761 | -0.000641 [-0.000921, 0.000157] | -0.001373 to 0.001004 |

The single five-fold feature-reduction analysis identified eight predictors as the smallest near-optimal XGBoost panel. Because the adjacent nine-predictor panel showed similar performance, the two panels were compared using 10 repeats of paired stratified five-fold cross-validation (seeds 42-51) with identical fold assignments. Each repeat metric was pooled across held-out participants. Compared with eight predictors, nine predictors improved AUPRC in 9/10 repeats, AUROC in 10/10, and Brier score in 7/10. Repeats are correlated assessments on the same participants, not independent cohorts. The nine-predictor panel showed small but directionally consistent incremental performance and was retained as the final model. This was an added development-stage stability check, not a study-start prespecified analysis. The reviewed implementation did not fit or tune the panels using external outcomes; historical external results had been seen, so complete historical blinding is not claimed. Age; sex; eGFRcr; HGB; PLT; BUN; ALB; ALP; GLU. The 8-variable panel contained the same predictors except ALP. The final XGBoost hyperparameters were those in Table S3 and were not retuned during this comparison.

## Table S5A Original-probability model performance

| **Cohort** | **Model** | **AUROC (95% CI)** | **AUPRC (95% CI)** | **Brier (95% CI)** |
| --- | --- | --- | --- | --- |
| Development | XGBoost | 0.863 (0.833, 0.889) | 0.600 (0.532, 0.668) | 0.102 (0.091, 0.115) |
| Development | Clinical core | 0.791 (0.755, 0.823) | 0.461 (0.400, 0.536) | 0.122 (0.109, 0.134) |
| Development | eGFRcr-only LR | 0.818 (0.785, 0.847) | 0.432 (0.378, 0.494) | 0.134 (0.121, 0.148) |
| Development | eGFRcr + BUN + ALB LR | 0.799 (0.765, 0.831) | 0.445 (0.387, 0.514) | 0.127 (0.114, 0.140) |
| Development | Rounded hand score | 0.799 (0.765, 0.831) | 0.445 (0.387, 0.513) | 0.127 (0.114, 0.140) |
| External | XGBoost | 0.806 (0.769, 0.840) | 0.258 (0.193, 0.337) | 0.144 (0.135, 0.153) |
| External | Clinical core | 0.661 (0.605, 0.715) | 0.126 (0.096, 0.176) | 0.130 (0.122, 0.140) |
| External | eGFRcr-only LR | 0.606 (0.552, 0.656) | 0.086 (0.069, 0.106) | 0.132 (0.122, 0.142) |
| External | eGFRcr + BUN + ALB LR | 0.695 (0.649, 0.737) | 0.116 (0.093, 0.147) | 0.151 (0.141, 0.160) |
| External | Rounded hand score | 0.695 (0.649, 0.737) | 0.116 (0.093, 0.147) | 0.151 (0.141, 0.160) |

Development: n=1170, D2=214, pooled OOF/model-selection performance. External: n=1619, D2=112. AP is average precision. Intervals: 2000 paired patient bootstrap resamples; no retraining. The additional LR comparators and rounded continuous hand score were evaluated as secondary analyses and did not inform final model selection.

## Table S5B Paired performance differences

| **Cohort** | **XGBoost minus comparator** | **Metric** | **Difference (95% CI)** |
| --- | --- | --- | --- |
| Development | XGBoost minus clinical core | AUROC | 0.073 (0.044, 0.103) |
| Development | XGBoost minus clinical core | AUPRC | 0.139 (0.073, 0.201) |
| Development | XGBoost minus clinical core | Brier | -0.019 (-0.028, -0.011) |
| Development | XGBoost minus eGFRcr-only LR | AUROC | 0.045 (0.025, 0.066) |
| Development | XGBoost minus eGFRcr-only LR | AUPRC | 0.168 (0.110, 0.219) |
| Development | XGBoost minus eGFRcr-only LR | Brier | -0.032 (-0.042, -0.022) |
| Development | XGBoost minus three-variable LR | AUROC | 0.064 (0.038, 0.093) |
| Development | XGBoost minus three-variable LR | AUPRC | 0.155 (0.094, 0.210) |
| Development | XGBoost minus three-variable LR | Brier | -0.024 (-0.034, -0.016) |
| External | XGBoost minus clinical core | AUROC | 0.145 (0.104, 0.187) |
| External | XGBoost minus clinical core | AUPRC | 0.132 (0.067, 0.198) |
| External | XGBoost minus clinical core | Brier | 0.014 (0.005, 0.022) |
| External | XGBoost minus eGFRcr-only LR | AUROC | 0.200 (0.158, 0.244) |
| External | XGBoost minus eGFRcr-only LR | AUPRC | 0.172 (0.113, 0.244) |
| External | XGBoost minus eGFRcr-only LR | Brier | 0.012 (0.001, 0.023) |
| External | XGBoost minus three-variable LR | AUROC | 0.111 (0.076, 0.147) |
| External | XGBoost minus three-variable LR | AUPRC | 0.142 (0.084, 0.210) |
| External | XGBoost minus three-variable LR | Brier | -0.007 (-0.017, 0.003) |

2,000 paired patient bootstrap resamples conditional on saved predictions. Lower Brier is better. Additional comparisons are exploratory.

## Table S5C Simple model coefficients

| **Model** | **Term** | **Coefficient original units** | **Three significant digits** | **Development median** |
| --- | --- | --- | --- | --- |
| eGFRcr-only LR | Intercept | 1.919773 | 1.920000 | NA |
| eGFRcr-only LR | eGFRcr_2021 | -0.034542 | -0.034500 | 107.9324 |
| eGFRcr + BUN + ALB LR | Intercept | 7.329178 | 7.330000 | NA |
| eGFRcr + BUN + ALB LR | eGFRcr_2021 | -0.040225 | -0.040200 | 107.9324 |
| eGFRcr + BUN + ALB LR | BUN | -0.084479 | -0.084500 | 5.9300 |
| eGFRcr + BUN + ALB LR | ALB | -0.100973 | -0.101000 | 43.6000 |

p=expit(intercept+sum coefficient×variable). eGFRcr: mL/min/1.73 m²; BUN: mmol/L; ALB: g/L. Only development data fitted the equations; imputation medians are fixed on development. The rounded three-variable linear score is hand-calculable and contains no external-optimized clinical thresholds.

## Table S5D Descriptive specificity at fixed sensitivity

| **Cohort** | **Model** | **Target sensitivity %** | **Achieved sensitivity %** | **Specificity %** |
| --- | --- | --- | --- | --- |
| Development | XGBoost | 80.0 | 80.4 | 78.6 |
| Development | XGBoost | 90.0 | 90.2 | 67.4 |
| Development | Clinical core | 80.0 | 80.4 | 59.3 |
| Development | Clinical core | 90.0 | 90.2 | 37.7 |
| Development | eGFRcr-only LR | 80.0 | 80.4 | 72.9 |
| Development | eGFRcr-only LR | 90.0 | 90.2 | 52.1 |
| Development | eGFRcr + BUN + ALB LR | 80.0 | 80.4 | 64.0 |
| Development | eGFRcr + BUN + ALB LR | 90.0 | 90.2 | 42.7 |
| External | XGBoost | 80.0 | 80.4 | 70.0 |
| External | XGBoost | 90.0 | 90.2 | 52.3 |
| External | Clinical core | 80.0 | 80.4 | 35.4 |
| External | Clinical core | 90.0 | 90.2 | 22.0 |
| External | eGFRcr-only LR | 80.0 | 80.4 | 40.3 |
| External | eGFRcr-only LR | 90.0 | 90.2 | 16.5 |
| External | eGFRcr + BUN + ALB LR | 80.0 | 80.4 | 53.2 |
| External | eGFRcr + BUN + ALB LR | 90.0 | 90.2 | 34.4 |

Each cohort uses its own empirical ROC point with achieved sensitivity at least the stated target. These are descriptive points, not development-derived probability cutoffs transported externally. Specificity can still depend on case mix.

## Table S6A Original and locally recalibrated metrics

| **Cohort** | **Model** | **Brier** | **Intercept** | **Slope** | **CITL** | **Mean p %** | **Observed %** |
| --- | --- | --- | --- | --- | --- | --- | --- |
| Development | XGBoost | 0.102 | -0.036 | 0.936 | 0.029 | 18.0 | 18.3 |
| Development | Clinical core | 0.122 | -0.019 | 0.999 | -0.018 | 18.5 | 18.3 |
| Development | eGFRcr-only LR | 0.134 | -0.026 | 0.982 | -0.001 | 18.3 | 18.3 |
| Development | Three-variable LR | 0.127 | -0.042 | 0.966 | 0.003 | 18.3 | 18.3 |
| Development | Rounded hand score | 0.127 | -0.043 | 0.965 | 0.004 | 18.2 | 18.3 |
| External | XGBoost | 0.144 | -2.209 | 0.848 | -2.250 | 29.9 | 6.9 |
| External | Clinical core | 0.130 | -2.150 | 0.441 | -1.916 | 26.7 | 6.9 |
| External | eGFRcr-only LR | 0.132 | -2.418 | 0.159 | -1.852 | 25.9 | 6.9 |
| External | Three-variable LR | 0.151 | -2.284 | 0.444 | -2.212 | 30.7 | 6.9 |
| External | Rounded hand score | 0.151 | -2.284 | 0.444 | -2.213 | 30.7 | 6.9 |
| External | Local crossfit XGBoost | 0.058 | -0.039 | 0.981 | -0.001 | 6.9 | 6.9 |

Development uses pooled OOF original probabilities. External original-probability rows apply development-fitted models unchanged. Local crossfit XGBoost is within-external-cohort adaptation sensitivity, not independent external validation. Intercept and slope are jointly estimated; CITL fixes slope at 1.

## Table S6B External local recalibration parameters

| **Fold** | **Training n/events** | **Held-out n/events** | **α** | **β** |
| --- | --- | --- | --- | --- |
| 1 | 1295/90 | 324/22 | -2.171845 | 0.795632 |
| 2 | 1295/90 | 324/22 | -2.232336 | 0.830022 |
| 3 | 1295/89 | 324/23 | -2.220411 | 0.830383 |
| 4 | 1295/89 | 324/23 | -2.200353 | 0.853255 |
| 5 | 1296/90 | 323/22 | -2.229067 | 0.939864 |

Five stratified folds, random_state = 42. logit(p_local) = alpha + beta x logit(p_original). Each participant received one held-out locally recalibrated probability; XGBoost was not retrained. Primary ROC, precision–recall, decision-curve, and targeted-testing results used the original model probabilities; local-recalibration sensitivity analyses are reported separately.

## Table S6C Pointwise external DCA sensitivity

| **Model** | **Threshold %** | **Net benefit (95% CI)** | **Test all NB** |
| --- | --- | --- | --- |
| XGBoost | 1 | 0.0600 (0.0481, 0.0730) | 0.0598 |
| XGBoost | 3 | 0.0450 (0.0329, 0.0582) | 0.0404 |
| XGBoost | 5 | 0.0311 (0.0189, 0.0446) | 0.0202 |
| XGBoost | 7 | 0.0181 (0.0058, 0.0311) | -0.0009 |
| XGBoost | 10 | -0.0007 (-0.0128, 0.0129) | -0.0342 |
| XGBoost | 12 | -0.0122 (-0.0249, 0.0014) | -0.0578 |
| XGBoost | 20 | -0.0578 (-0.0716, -0.0432) | -0.1635 |
| XGBoost | 30 | -0.1000 (-0.1163, -0.0838) | -0.3297 |
| XGBoost | 50 | -0.1600 (-0.1822, -0.1384) | -0.8616 |
| Clinical core | 1 | 0.0593 (0.0474, 0.0723) | 0.0598 |
| Clinical core | 3 | 0.0411 (0.0289, 0.0538) | 0.0404 |
| Clinical core | 5 | 0.0230 (0.0107, 0.0358) | 0.0202 |
| Clinical core | 7 | 0.0058 (-0.0064, 0.0187) | -0.0009 |
| Clinical core | 10 | -0.0147 (-0.0270, -0.0019) | -0.0342 |
| Clinical core | 12 | -0.0293 (-0.0413, -0.0162) | -0.0578 |
| Clinical core | 20 | -0.0656 (-0.0789, -0.0525) | -0.1635 |
| Clinical core | 30 | -0.0954 (-0.1103, -0.0809) | -0.3297 |
| Clinical core | 50 | -0.1062 (-0.1242, -0.0883) | -0.8616 |
| Local crossfit XGBoost | 1 | 0.0604 (0.0486, 0.0728) | 0.0598 |
| Local crossfit XGBoost | 3 | 0.0485 (0.0370, 0.0607) | 0.0404 |
| Local crossfit XGBoost | 5 | 0.0384 (0.0272, 0.0501) | 0.0202 |
| Local crossfit XGBoost | 7 | 0.0340 (0.0228, 0.0455) | -0.0009 |
| Local crossfit XGBoost | 10 | 0.0233 (0.0128, 0.0338) | -0.0342 |
| Local crossfit XGBoost | 12 | 0.0170 (0.0077, 0.0260) | -0.0578 |
| Local crossfit XGBoost | 20 | 0.0063 (-0.0008, 0.0134) | -0.1635 |
| Local crossfit XGBoost | 30 | 0.0016 (-0.0036, 0.0071) | -0.3297 |
| Local crossfit XGBoost | 50 | 0.0000 (-0.0025, 0.0025) | -0.8616 |

Exploratory fixed thresholds; no established project-specific clinical threshold. Intervals condition on saved predictions; local recalibrator not refitted within bootstrap. Local versus unadapted models is not a fair original-model comparison. Test-none net benefit=0.

## Table S7A Development fixed-budget comparisons

| **Strategy** | **Budget %** | **Tested n** | **D2 n** | **Recall % (95% CI)** | **Yield % (95% CI)** | **Tests per case (95% CI)** | **Enrichment (95% CI)** |
| --- | --- | --- | --- | --- | --- | --- | --- |
| XGBoost | 10 | 117 | 80 | 37.4 (32.7, 42.5) | 68.4 (59.8, 76.9) | 1.462 (1.300, 1.671) | 3.738 (3.274, 4.253) |
| XGBoost | 20 | 234 | 131 | 61.2 (55.7, 67.5) | 56.0 (49.1, 63.2) | 1.786 (1.581, 2.035) | 3.061 (2.786, 3.374) |
| XGBoost | 30 | 351 | 168 | 78.5 (73.4, 83.7) | 47.9 (41.9, 53.8) | 2.089 (1.857, 2.388) | 2.617 (2.447, 2.788) |
| XGBoost | 40 | 468 | 186 | 86.9 (82.4, 91.3) | 39.7 (35.0, 44.7) | 2.516 (2.239, 2.854) | 2.173 (2.059, 2.283) |
| XGBoost | 50 | 585 | 198 | 92.5 (88.4, 95.7) | 33.8 (29.6, 38.1) | 2.955 (2.623, 3.382) | 1.850 (1.768, 1.913) |
| Clinical core | 10 | 117 | 66 | 30.8 (26.8, 36.0) | 56.4 (47.9, 66.7) | 1.773 (1.500, 2.089) | 3.084 (2.679, 3.598) |
| Clinical core | 20 | 234 | 114 | 53.3 (47.2, 59.2) | 48.7 (41.9, 55.1) | 2.053 (1.814, 2.388) | 2.664 (2.360, 2.961) |
| Clinical core | 30 | 351 | 147 | 68.7 (63.2, 74.6) | 41.9 (36.5, 47.9) | 2.388 (2.089, 2.742) | 2.290 (2.107, 2.488) |
| Clinical core | 40 | 468 | 165 | 77.1 (71.6, 82.4) | 35.3 (30.8, 40.2) | 2.836 (2.489, 3.250) | 1.928 (1.791, 2.060) |
| Clinical core | 50 | 585 | 176 | 82.2 (76.9, 86.8) | 30.1 (26.2, 34.0) | 3.324 (2.939, 3.824) | 1.645 (1.538, 1.736) |
| eGFRcr-only LR | 10 | 117 | 61 | 28.5 (23.5, 33.5) | 52.1 (42.7, 60.7) | 1.918 (1.648, 2.340) | 2.850 (2.345, 3.350) |
| eGFRcr-only LR | 20 | 234 | 123 | 57.5 (51.0, 63.2) | 52.6 (45.7, 58.5) | 1.902 (1.708, 2.187) | 2.874 (2.551, 3.159) |
| eGFRcr-only LR | 30 | 351 | 157 | 73.4 (68.1, 78.9) | 44.7 (38.7, 50.7) | 2.236 (1.972, 2.581) | 2.445 (2.271, 2.630) |
| eGFRcr-only LR | 40 | 468 | 177 | 82.7 (77.6, 87.4) | 37.8 (33.1, 42.7) | 2.644 (2.340, 3.019) | 2.068 (1.940, 2.185) |
| eGFRcr-only LR | 50 | 585 | 185 | 86.4 (81.5, 90.9) | 31.6 (27.5, 36.1) | 3.162 (2.773, 3.634) | 1.729 (1.630, 1.817) |
| Three-variable LR | 10 | 117 | 65 | 30.4 (25.2, 35.2) | 55.6 (46.2, 64.1) | 1.800 (1.560, 2.167) | 3.037 (2.522, 3.519) |
| Three-variable LR | 20 | 234 | 127 | 59.3 (53.8, 65.1) | 54.3 (47.4, 61.1) | 1.843 (1.636, 2.108) | 2.967 (2.689, 3.255) |
| Three-variable LR | 30 | 351 | 151 | 70.6 (64.5, 75.7) | 43.0 (37.3, 48.7) | 2.325 (2.053, 2.680) | 2.352 (2.148, 2.524) |
| Three-variable LR | 40 | 468 | 163 | 76.2 (70.7, 81.5) | 34.8 (30.6, 39.3) | 2.871 (2.543, 3.273) | 1.904 (1.768, 2.038) |
| Three-variable LR | 50 | 585 | 178 | 83.2 (77.9, 87.7) | 30.4 (26.3, 34.4) | 3.287 (2.910, 3.799) | 1.664 (1.559, 1.753) |
| Rounded hand score | 10 | 117 | 65 | 30.4 (25.2, 35.2) | 55.6 (46.2, 64.1) | 1.800 (1.560, 2.167) | 3.037 (2.521, 3.518) |
| Rounded hand score | 20 | 234 | 127 | 59.3 (53.7, 65.0) | 54.3 (47.4, 61.1) | 1.843 (1.636, 2.108) | 2.967 (2.683, 3.252) |
| Rounded hand score | 30 | 351 | 150 | 70.1 (64.5, 75.8) | 42.7 (37.3, 48.7) | 2.340 (2.053, 2.679) | 2.336 (2.150, 2.528) |
| Rounded hand score | 40 | 468 | 163 | 76.2 (70.7, 81.3) | 34.8 (30.6, 39.3) | 2.871 (2.543, 3.273) | 1.904 (1.767, 2.033) |
| Rounded hand score | 50 | 585 | 178 | 83.2 (78.1, 87.7) | 30.4 (26.3, 34.5) | 3.287 (2.896, 3.799) | 1.664 (1.562, 1.755) |

Each strategy scored the same study cohort under the same testing budget; rankings and selected individuals could differ. Counts=floor(N×allocation). Original model probabilities, or the development-fitted rounded score, were used. Intervals use 2,000 paired patient bootstrap resamples with reranking. Additional comparisons are exploratory.

## Table S7B External fixed-budget comparisons

| **Strategy** | **Budget %** | **Tested n** | **D2 n** | **Recall % (95% CI)** | **Yield % (95% CI)** | **Tests per case (95% CI)** | **Enrichment (95% CI)** |
| --- | --- | --- | --- | --- | --- | --- | --- |
| XGBoost | 10 | 161 | 39 | 34.8 (27.2, 43.4) | 24.2 (18.0, 31.1) | 4.128 (3.220, 5.552) | 3.502 (2.734, 4.369) |
| XGBoost | 20 | 323 | 62 | 55.4 (47.5, 64.6) | 19.2 (15.5, 23.5) | 5.210 (4.250, 6.460) | 2.775 (2.382, 3.240) |
| XGBoost | 30 | 485 | 82 | 73.2 (65.3, 81.3) | 16.9 (13.6, 20.2) | 5.915 (4.949, 7.348) | 2.444 (2.181, 2.715) |
| XGBoost | 40 | 647 | 94 | 83.9 (76.5, 89.7) | 14.5 (11.7, 17.2) | 6.883 (5.829, 8.513) | 2.100 (1.914, 2.245) |
| XGBoost | 50 | 809 | 100 | 89.3 (83.0, 94.7) | 12.4 (10.3, 14.6) | 8.090 (6.856, 9.747) | 1.787 (1.661, 1.896) |
| Clinical core | 10 | 161 | 27 | 24.1 (15.8, 30.7) | 16.8 (10.6, 21.7) | 5.963 (4.600, 9.471) | 2.424 (1.588, 3.085) |
| Clinical core | 20 | 323 | 42 | 37.5 (29.4, 46.3) | 13.0 (9.6, 16.7) | 7.690 (5.981, 10.419) | 1.880 (1.472, 2.321) |
| Clinical core | 30 | 485 | 58 | 51.8 (43.4, 61.5) | 12.0 (9.3, 14.8) | 8.362 (6.736, 10.778) | 1.729 (1.448, 2.054) |
| Clinical core | 40 | 647 | 72 | 64.3 (55.4, 73.1) | 11.1 (8.8, 13.6) | 8.986 (7.352, 11.356) | 1.609 (1.387, 1.830) |
| Clinical core | 50 | 809 | 81 | 72.3 (63.5, 80.5) | 10.0 (8.0, 12.0) | 9.988 (8.340, 12.446) | 1.447 (1.270, 1.611) |
| eGFRcr-only LR | 10 | 161 | 9 | 8.0 (3.4, 13.5) | 5.6 (2.5, 9.3) | 17.889 (10.733, 40.250) | 0.808 (0.347, 1.357) |
| eGFRcr-only LR | 20 | 323 | 30 | 26.8 (18.4, 34.7) | 9.3 (6.2, 12.4) | 10.767 (8.075, 16.150) | 1.343 (0.921, 1.740) |
| eGFRcr-only LR | 30 | 485 | 47 | 42.0 (33.6, 51.3) | 9.7 (7.2, 12.4) | 10.319 (8.083, 13.857) | 1.401 (1.123, 1.713) |
| eGFRcr-only LR | 40 | 647 | 64 | 57.1 (48.6, 66.4) | 9.9 (7.7, 12.4) | 10.109 (8.088, 12.940) | 1.430 (1.217, 1.660) |
| eGFRcr-only LR | 50 | 809 | 80 | 71.4 (63.8, 80.0) | 9.9 (7.9, 12.0) | 10.113 (8.340, 12.641) | 1.429 (1.277, 1.601) |
| Three-variable LR | 10 | 161 | 20 | 17.9 (11.2, 24.5) | 12.4 (7.5, 17.4) | 8.050 (5.750, 13.417) | 1.796 (1.127, 2.468) |
| Three-variable LR | 20 | 323 | 43 | 38.4 (30.0, 47.0) | 13.3 (9.6, 16.7) | 7.512 (5.981, 10.419) | 1.924 (1.504, 2.356) |
| Three-variable LR | 30 | 485 | 65 | 58.0 (49.1, 66.9) | 13.4 (10.5, 16.3) | 7.462 (6.137, 9.515) | 1.937 (1.639, 2.234) |
| Three-variable LR | 40 | 647 | 76 | 67.9 (59.8, 76.4) | 11.7 (9.4, 14.4) | 8.513 (6.957, 10.607) | 1.698 (1.497, 1.911) |
| Three-variable LR | 50 | 809 | 91 | 81.2 (73.6, 88.2) | 11.2 (9.1, 13.5) | 8.890 (7.422, 10.932) | 1.626 (1.473, 1.765) |
| Rounded hand score | 10 | 161 | 20 | 17.9 (11.2, 24.5) | 12.4 (7.5, 17.4) | 8.050 (5.750, 13.417) | 1.796 (1.129, 2.468) |
| Rounded hand score | 20 | 323 | 43 | 38.4 (29.9, 47.0) | 13.3 (9.6, 16.7) | 7.512 (5.981, 10.419) | 1.924 (1.499, 2.356) |
| Rounded hand score | 30 | 485 | 65 | 58.0 (49.2, 66.9) | 13.4 (10.5, 16.3) | 7.462 (6.137, 9.515) | 1.937 (1.641, 2.234) |
| Rounded hand score | 40 | 647 | 76 | 67.9 (59.8, 76.4) | 11.7 (9.4, 14.4) | 8.513 (6.957, 10.607) | 1.698 (1.497, 1.911) |
| Rounded hand score | 50 | 809 | 91 | 81.2 (73.6, 88.2) | 11.2 (9.1, 13.5) | 8.890 (7.422, 10.932) | 1.626 (1.473, 1.765) |

Each strategy scored the same study cohort under the same testing budget; rankings and selected individuals could differ. Counts=floor(N×allocation). Original model probabilities, or the development-fitted rounded score, were used. Intervals use 2,000 paired patient bootstrap resamples with reranking. Additional comparisons are exploratory.

## Table S7C Paired recall increments across fixed budgets

| **Cohort** | **XGBoost minus comparator** | **Budget %** | **Recall difference pp (95% CI)** |
| --- | --- | --- | --- |
| Development | XGBoost minus clinical core | 10 | 6.5 (0.9, 11.4) |
| Development | XGBoost minus clinical core | 20 | 7.9 (2.9, 14.1) |
| Development | XGBoost minus clinical core | 30 | 9.8 (4.5, 14.7) |
| Development | XGBoost minus clinical core | 40 | 9.8 (4.8, 15.2) |
| Development | XGBoost minus clinical core | 50 | 10.3 (5.7, 15.1) |
| Development | XGBoost minus eGFRcr-only LR | 10 | 8.9 (3.7, 14.8) |
| Development | XGBoost minus eGFRcr-only LR | 20 | 3.7 (-0.5, 9.6) |
| Development | XGBoost minus eGFRcr-only LR | 30 | 5.1 (0.4, 9.4) |
| Development | XGBoost minus eGFRcr-only LR | 40 | 4.2 (0.0, 8.7) |
| Development | XGBoost minus eGFRcr-only LR | 50 | 6.1 (2.4, 9.4) |
| Development | XGBoost minus three-variable LR | 10 | 7.0 (2.3, 12.4) |
| Development | XGBoost minus three-variable LR | 20 | 1.9 (-2.7, 6.9) |
| Development | XGBoost minus three-variable LR | 30 | 7.9 (3.1, 13.2) |
| Development | XGBoost minus three-variable LR | 40 | 10.7 (5.9, 15.9) |
| Development | XGBoost minus three-variable LR | 50 | 9.3 (4.6, 13.9) |

## Table S7C Paired recall increments across fixed budgets continued

| **Cohort** | **XGBoost minus comparator** | **Budget %** | **Recall difference pp (95% CI)** |
| --- | --- | --- | --- |
| External | XGBoost minus clinical core | 10 | 10.7 (2.5, 21.6) |
| External | XGBoost minus clinical core | 20 | 17.9 (9.8, 27.0) |
| External | XGBoost minus clinical core | 30 | 21.4 (12.7, 29.4) |
| External | XGBoost minus clinical core | 40 | 19.6 (11.3, 27.3) |
| External | XGBoost minus clinical core | 50 | 17.0 (9.2, 25.6) |
| External | XGBoost minus eGFRcr-only LR | 10 | 26.8 (16.9, 36.9) |
| External | XGBoost minus eGFRcr-only LR | 20 | 28.6 (18.9, 40.0) |
| External | XGBoost minus eGFRcr-only LR | 30 | 31.2 (21.1, 40.6) |
| External | XGBoost minus eGFRcr-only LR | 40 | 26.8 (17.1, 34.5) |
| External | XGBoost minus eGFRcr-only LR | 50 | 17.9 (10.5, 24.8) |
| External | XGBoost minus three-variable LR | 10 | 17.0 (8.0, 27.4) |
| External | XGBoost minus three-variable LR | 20 | 17.0 (8.6, 27.2) |
| External | XGBoost minus three-variable LR | 30 | 15.2 (6.7, 23.6) |
| External | XGBoost minus three-variable LR | 40 | 16.1 (8.2, 22.6) |
| External | XGBoost minus three-variable LR | 50 | 8.0 (0.9, 15.7) |

The same bootstrap draws were used for each contrast. Development intervals do not account for model-selection uncertainty. No multiplicity adjustment. The external 20% difference versus three-variable LR is +17.0 pp (95% CI 8.6–27.2); development is +1.9 pp (−2.7–6.9).

## Table S8A Development four-group profiles

| **Phenotype** | **D2− / Model−** | **D2− / Model+** | **D2+ / Model−** | **D2+ / Model+** |
| --- | --- | --- | --- | --- |
| Participants, n | 853 | 103 | 83 | 131 |
| D1, n/N (%) | 125/853 (14.65%) | 29/103 (28.16%) | 80/83 (96.39%) | 128/131 (97.71%) |
| D3, n/eligible N (%) | 2/837 (0.24%) | 8/76 (10.53%) | 31/76 (40.79%) | 89/105 (84.76%) |
| eGFRcys < 60, n/N (%) | 18/853 (2.11%) | 32/103 (31.07%) | 38/83 (45.78%) | 115/131 (87.79%) |
| eGFRcys | 112.62 [99.26, 124.61] | 71.82 [53.48, 89.68] | 60.75 [51.65, 72.41] | 43.19 [32.36, 54.55] |
| eGFR difference | 0.75 [-9.25, 9.34] | -7.78 [-16.05, -0.47] | -38.57 [-45.75, -34.37] | -36.55 [-43.26, -28.14] |
| Cys-C, mg/L | 0.69 [0.57, 0.81] | 0.99 [0.83, 1.27] | 1.18 [1.06, 1.33] | 1.47 [1.24, 1.82] |

Model-positive is the identical fixed top-20% XGBoost selection in Figure 5: 234 development and 323 external participants. D3 denominators include only participants with eGFRcr ≥60 mL/min/1.73 m². Continuous values are medians [Q1, Q3]; eGFR and difference units are mL/min/1.73 m². The prespecified primary descriptive contrast is D2-negative/model-positive versus D2-negative/model-negative; the remaining groups provide context. These related cystatin C-based phenotypes are not independent disease diagnoses.

## Table S8B External four-group profiles

| **Phenotype** | **D2− / Model−** | **D2− / Model+** | **D2+ / Model−** | **D2+ / Model+** |
| --- | --- | --- | --- | --- |
| Participants, n | 1246 | 261 | 50 | 62 |
| D1, n/N (%) | 100/1246 (8.03%) | 52/261 (19.92%) | 46/50 (92.00%) | 61/62 (98.39%) |
| D3, n/eligible N (%) | 18/1094 (1.65%) | 25/191 (13.09%) | 21/43 (48.84%) | 41/53 (77.36%) |
| eGFRcys < 60, n/N (%) | 151/1246 (12.12%) | 85/261 (32.57%) | 28/50 (56.00%) | 50/62 (80.65%) |
| eGFRcys | 103.68 [82.80, 115.18] | 70.89 [55.83, 86.70] | 57.46 [45.88, 66.14] | 46.28 [36.22, 55.97] |
| eGFR difference | 2.64 [-4.46, 8.84] | -3.28 [-11.67, 3.60] | -37.29 [-53.13, -29.45] | -31.03 [-36.96, -25.88] |
| Cys-C, mg/L | 0.76 [0.67, 0.93] | 1.00 [0.88, 1.22] | 1.23 [1.09, 1.43] | 1.35 [1.17, 1.67] |

Model-positive is the identical fixed top-20% XGBoost selection in Figure 5: 234 development and 323 external participants. D3 denominators include only participants with eGFRcr ≥60 mL/min/1.73 m². Continuous values are medians [Q1, Q3]; eGFR and difference units are mL/min/1.73 m². The prespecified primary descriptive contrast is D2-negative/model-positive versus D2-negative/model-negative; the remaining groups provide context. These related cystatin C-based phenotypes are not independent disease diagnoses.

## Table S9A Binary phenotype contrasts

| **Cohort** | **Phenotype** | **Model+ n/N (%)** | **Model− n/N (%)** | **Absolute RD, pp (95% CI)** | **RR (95% CI)** |
| --- | --- | --- | --- | --- | --- |
| Development cohort | D1 | 29/103 (28.16%) | 125/853 (14.65%) | 13.50 (5.32, 23.11) | 1.92 (1.36, 2.72) |
| Development cohort | D3 | 8/76 (10.53%) | 2/837 (0.24%) | 10.29 (5.15, 19.18) | 44.05 (9.52, 203.77) |
| Development cohort | eGFRcys < 60 | 32/103 (31.07%) | 18/853 (2.11%) | 28.96 (20.75, 38.47) | 14.72 (8.58, 25.27) |
| External validation cohort | D1 | 52/261 (19.92%) | 100/1246 (8.03%) | 11.90 (7.21, 17.34) | 2.48 (1.83, 3.38) |
| External validation cohort | D3 | 25/191 (13.09%) | 18/1094 (1.65%) | 11.44 (7.27, 17.00) | 7.96 (4.43, 14.29) |
| External validation cohort | eGFRcys < 60 | 85/261 (32.57%) | 151/1246 (12.12%) | 20.45 (14.72, 26.59) | 2.69 (2.14, 3.38) |

Contrast: Model+ minus Model- among D2-negative participants. Absolute risk differences (RD), in percentage points (pp), are the primary effect measure, with Newcombe 95% confidence intervals. Risk ratios (RR) use log intervals and are secondary. The development D3 reference group contains only two events, so the absolute difference and its uncertainty should be emphasized over the RR. D3 denominators are restricted to participants with eGFRcr ≥60 mL/min/1.73 m². These comparisons are descriptive and not multiplicity-adjusted.

## Table S9B Continuous phenotype contrasts

| **Cohort** | **Phenotype** | **Model+ median** | **Model− median** | **Median difference (95% CI)** |
| --- | --- | --- | --- | --- |
| Development cohort | eGFRcys_2012 | 71.82 | 112.62 | -40.80 (-45.08, -34.33) |
| Development cohort | eGFR_difference | -7.78 | 0.75 | -8.53 (-12.55, -5.17) |
| Development cohort | CysC_mg_L | 0.99 | 0.69 | 0.30 (0.27, 0.36) |
| External validation cohort | eGFRcys_2012 | 70.89 | 103.68 | -32.78 (-35.77, -29.38) |
| External validation cohort | eGFR_difference | -3.28 | 2.64 | -5.92 (-7.74, -4.79) |
| External validation cohort | CysC_mg_L | 1.00 | 0.76 | 0.24 (0.21, 0.27) |

Differences are Model+ minus Model− among D2-negative participants. eGFR and difference units are mL/min/1.73 m²; cystatin C units are mg/L. Percentile 95% confidence intervals use 2,000 independent within-group patient resamples with group membership fixed, without model refitting or reranking. Intervals are descriptive and not multiplicity-adjusted. The ratio defining D2 is not included as an additional continuous contrast.

## Table S10A Development included and excluded

| **Variable** | **Included median (Q1, Q3) or %** | **Valid/missing n (%)** | **Excluded median (Q1, Q3) or %** | **Valid/missing n (%)** | **SMD** |
| --- | --- | --- | --- | --- | --- |
| age | 57.00 (50.00, 66.00) | 1170/0 (0.0%) | 57.00 (49.00, 68.00) | 842/0 (0.0%) | -0.045 |
| sex | 59.1% | 1170/0 (0.0%) | 58.7% | 842/0 (0.0%) | 0.008 |
| eGFRcr_2021 | 107.93 (98.19, 116.20) | 1170/0 (0.0%) | 106.92 (94.40, 116.11) | 842/0 (0.0%) | 0.097 |
| CysC_mg_L | 0.77 (0.61, 1.01) | 1170/0 (0.0%) | 0.77 (0.60, 1.03) | 842/0 (0.0%) | -0.019 |
| SCr_mg_dL | 0.62 (0.52, 0.76) | 1170/0 (0.0%) | 0.64 (0.53, 0.79) | 842/0 (0.0%) | -0.052 |
| eGFRcys_2012 | 104.10 (73.96, 120.25) | 1170/0 (0.0%) | 103.93 (72.16, 123.45) | 842/0 (0.0%) | -0.017 |
| D2 | 18.3% | 1170/0 (0.0%) | 17.8% | 842/0 (0.0%) | 0.012 |
| BUN | 5.93 (4.89, 7.38) | 1170/0 (0.0%) | 5.92 (4.83, 7.32) | 842/0 (0.0%) | -0.014 |
| ALB | 43.60 (40.70, 46.10) | 1150/20 (1.7%) | 43.30 (40.00, 46.00) | 819/23 (2.7%) | 0.061 |
| WBC | 6.19 (5.10, 7.60) | 1170/0 (0.0%) | NA (NA, NA) | 0/842 (100.0%) | NA |
| HGB | 136.00 (122.00, 147.00) | 1170/0 (0.0%) | NA (NA, NA) | 0/842 (100.0%) | NA |
| PLT | 217.00 (178.00, 263.00) | 1170/0 (0.0%) | NA (NA, NA) | 0/842 (100.0%) | NA |
| ALP | 82.00 (69.00, 101.00) | 1150/20 (1.7%) | 84.00 (68.00, 101.00) | 819/23 (2.7%) | -0.025 |
| GLU | 9.30 (7.01, 12.37) | 1136/34 (2.9%) | 9.31 (6.98, 12.38) | 810/32 (3.8%) | 0.032 |
| HbA1c | 9.05 (7.50, 11.10) | 972/198 (16.9%) | 9.10 (7.60, 10.90) | 692/150 (17.8%) | 0.052 |
| TC | 4.39 (3.68, 5.11) | 1143/27 (2.3%) | 4.39 (3.64, 5.15) | 812/30 (3.6%) | 0.037 |
| HDL-C | 1.00 (0.85, 1.18) | 1146/24 (2.1%) | 0.91 (0.75, 1.12) | 812/30 (3.6%) | 0.263 |
| TG | 1.61 (1.12, 2.50) | 1146/24 (2.1%) | 1.50 (1.04, 2.31) | 812/30 (3.6%) | 0.040 |

Continuous SMD uses mean differences divided by the square root of average sample variances; binary SMD uses pooled Bernoulli variance. Sex=% male. Missingness is not imputed. The invalid CBC case is treated as module-missing only in the descriptive copy. NA indicates insufficient valid data. eGFR mL/min/1.73 m²; SCr mg/dL; CysC mg/L; BUN/GLU/TC/HDL-C/TG mmol/L; ALB/HGB g/L; PLT/WBC 10⁹/L; ALP U/L; HbA1c %.

## Table S10B External included and excluded

| **Variable** | **Included median (Q1, Q3) or %** | **Valid/missing n (%)** | **Excluded median (Q1, Q3) or %** | **Valid/missing n (%)** | **SMD** |
| --- | --- | --- | --- | --- | --- |
| age | 61.00 (52.00, 70.00) | 1619/0 (0.0%) | 59.00 (51.75, 68.00) | 156/0 (0.0%) | 0.181 |
| sex | 65.3% | 1619/0 (0.0%) | 70.5% | 156/0 (0.0%) | -0.112 |
| eGFRcr_2021 | 97.89 (78.98, 108.17) | 1619/0 (0.0%) | 89.81 (47.68, 106.93) | 156/0 (0.0%) | 0.405 |
| CysC_mg_L | 0.82 (0.69, 1.06) | 1619/0 (0.0%) | 0.99 (0.70, 1.58) | 156/0 (0.0%) | -0.396 |
| SCr_mg_dL | 0.77 (0.62, 0.98) | 1619/0 (0.0%) | 0.94 (0.66, 1.57) | 156/0 (0.0%) | -0.400 |
| eGFRcys_2012 | 96.09 (68.02, 111.81) | 1619/0 (0.0%) | 74.19 (40.71, 110.94) | 156/0 (0.0%) | 0.339 |
| D2 | 6.9% | 1619/0 (0.0%) | 7.7% | 156/0 (0.0%) | -0.030 |
| BUN | 5.78 (4.58, 7.50) | 1619/0 (0.0%) | 6.32 (4.99, 9.16) | 156/0 (0.0%) | -0.271 |
| ALB | 41.25 (37.30, 44.20) | 1552/67 (4.1%) | 42.75 (38.83, 45.95) | 110/46 (29.5%) | -0.234 |
| WBC | 6.29 (5.17, 7.82) | 1619/0 (0.0%) | NA (NA, NA) | 0/156 (100.0%) | NA |
| HGB | 132.00 (116.00, 145.00) | 1619/0 (0.0%) | 141.00 (141.00, 141.00) | 1/155 (99.4%) | NA |
| PLT | 203.00 (164.00, 253.50) | 1619/0 (0.0%) | 192.00 (192.00, 192.00) | 1/155 (99.4%) | NA |
| ALP | 77.00 (63.00, 97.00) | 1552/67 (4.1%) | 79.00 (63.25, 95.75) | 110/46 (29.5%) | 0.080 |
| GLU | 7.53 (5.99, 9.88) | 1520/99 (6.1%) | 7.07 (5.79, 8.98) | 102/54 (34.6%) | 0.009 |
| HbA1c | NA (NA, NA) | 0/1619 (100.0%) | NA (NA, NA) | 0/156 (100.0%) | NA |
| TC | 4.22 (3.53, 4.98) | 934/685 (42.3%) | 4.28 (3.42, 4.94) | 79/77 (49.4%) | 0.014 |
| HDL-C | 1.05 (0.87, 1.25) | 934/685 (42.3%) | 1.04 (0.88, 1.27) | 79/77 (49.4%) | -0.020 |
| TG | 1.41 (0.96, 2.12) | 934/685 (42.3%) | 1.58 (0.95, 2.57) | 79/77 (49.4%) | -0.132 |

Continuous SMD uses mean differences divided by the square root of average sample variances; binary SMD uses pooled Bernoulli variance. Sex=% male. Missingness is not imputed. The invalid CBC case is treated as module-missing only in the descriptive copy. NA indicates insufficient valid data. eGFR mL/min/1.73 m²; SCr mg/dL; CysC mg/L; BUN/GLU/TC/HDL-C/TG mmol/L; ALB/HGB g/L; PLT/WBC 10⁹/L; ALP U/L; HbA1c %.

## Table S10C Between-center case mix

| **Variable** | **Development median (Q1, Q3) or %** | **Valid/missing n** | **External median (Q1, Q3) or %** | **Valid/missing n** | **SMD** |
| --- | --- | --- | --- | --- | --- |
| age | 57.00 (50.00, 66.00) | 1170/0 | 61.00 (52.00, 70.00) | 1619/0 | -0.266 |
| sex | 59.1% | 1170/0 | 65.3% | 1619/0 | -0.129 |
| eGFRcr_2021 | 107.93 (98.19, 116.20) | 1170/0 | 97.89 (78.98, 108.17) | 1619/0 | 0.502 |
| CysC_mg_L | 0.77 (0.61, 1.01) | 1170/0 | 0.82 (0.69, 1.06) | 1619/0 | -0.097 |
| SCr_mg_dL | 0.62 (0.52, 0.76) | 1170/0 | 0.77 (0.62, 0.98) | 1619/0 | -0.265 |
| eGFRcys_2012 | 104.10 (73.96, 120.25) | 1170/0 | 96.09 (68.02, 111.81) | 1619/0 | 0.251 |
| D2 | 18.3% | 1170/0 | 6.9% | 1619/0 | 0.348 |
| BUN | 5.93 (4.89, 7.38) | 1170/0 | 5.78 (4.58, 7.50) | 1619/0 | -0.029 |
| ALB | 43.60 (40.70, 46.10) | 1150/20 | 41.25 (37.30, 44.20) | 1552/67 | 0.527 |
| WBC | 6.19 (5.10, 7.60) | 1170/0 | 6.29 (5.17, 7.82) | 1619/0 | -0.060 |
| HGB | 136.00 (122.00, 147.00) | 1170/0 | 132.00 (116.00, 145.00) | 1619/0 | 0.213 |
| PLT | 217.00 (178.00, 263.00) | 1170/0 | 203.00 (164.00, 253.50) | 1619/0 | 0.147 |
| ALP | 82.00 (69.00, 101.00) | 1150/20 | 77.00 (63.00, 97.00) | 1552/67 | 0.035 |
| GLU | 9.30 (7.01, 12.37) | 1136/34 | 7.53 (5.99, 9.88) | 1520/99 | 0.473 |
| HbA1c | 9.05 (7.50, 11.10) | 972/198 | NA (NA, NA) | 0/1619 | NA |
| TC | 4.39 (3.68, 5.11) | 1143/27 | 4.22 (3.53, 4.98) | 934/685 | 0.137 |
| HDL-C | 1.00 (0.85, 1.18) | 1146/24 | 1.05 (0.87, 1.25) | 934/685 | -0.174 |
| TG | 1.61 (1.12, 2.50) | 1146/24 | 1.41 (0.96, 2.12) | 934/685 | 0.160 |

SMD=development minus external, using mean/SD for continuous and proportions for binary measures. eGFR: mL/min/1.73 m²; SCr: mg/dL; CysC: mg/L; BUN/GLU: mmol/L; ALB/HGB: g/L; PLT/WBC: 10^9/L; ALP: U/L. Observational differences do not establish causes of prevalence shift.

## Table S10D Cystatin C testing within the available extraction

| **Cohort** | **SCr eligible adults with diabetes** | **Ever had valid CysC n** | **Percentage** |
| --- | --- | --- | --- |
| Development | 2210 | 2012 | 91.0 |
| External | 2156 | 1775 | 82.3 |

Denominator is limited to the available extraction, not all adults with diabetes at either hospital. No complete, verifiable ordering-indication data are available. These figures do not establish institutional coverage or causal selection mechanisms.

## Table S11A Missingness of the nine frozen predictors

| **Cohort** | **Predictor** | **N** | **Missing n** | **Missing %** |
| --- | --- | --- | --- | --- |
| Development | age | 1170 | 0 | 0.00 |
| Development | sex | 1170 | 0 | 0.00 |
| Development | eGFRcr_2021 | 1170 | 0 | 0.00 |
| Development | HGB | 1170 | 0 | 0.00 |
| Development | PLT | 1170 | 0 | 0.00 |
| Development | BUN | 1170 | 0 | 0.00 |
| Development | ALB | 1170 | 20 | 1.71 |
| Development | ALP | 1170 | 20 | 1.71 |
| Development | GLU | 1170 | 34 | 2.91 |
| External | age | 1619 | 0 | 0.00 |
| External | sex | 1619 | 0 | 0.00 |
| External | eGFRcr_2021 | 1619 | 0 | 0.00 |
| External | HGB | 1619 | 0 | 0.00 |
| External | PLT | 1619 | 0 | 0.00 |
| External | BUN | 1619 | 0 | 0.00 |
| External | ALB | 1619 | 67 | 4.14 |
| External | ALP | 1619 | 67 | 4.14 |
| External | GLU | 1619 | 99 | 6.11 |

Observed input missingness before imputation. Age/sex/eGFRcr and eligibility-defining CBC fields are complete in the final cohorts. Median imputation was performed inside training folds; external used development-trained medians.

## Table S11B Missing-data sensitivity

| **Cohort** | **Analysis** | **N** | **D2 n** | **AUROC** | **AUPRC** | **Brier** |
| --- | --- | --- | --- | --- | --- | --- |
| Development | Complete-case frozen scores | 1135 | 201 | 0.861 | 0.586 | 0.101 |
| External | Complete-case frozen scores | 1513 | 102 | 0.812 | 0.258 | 0.141 |
| Development | Mean-imputation sensitivity | 1170 | 214 | 0.865 (0.834, 0.890) | 0.598 (0.528, 0.667) | 0.102 (0.091, 0.114) |
| External | Mean-imputation sensitivity | 1619 | 112 | 0.805 (0.768, 0.840) | 0.256 (0.192, 0.332) | 0.144 (0.135, 0.153) |

Complete cases retain original development OOF or frozen external scores, without refitting. Mean-imputation models refit only the original fixed XGBoost specification with an alternative imputer and the same folds. They do not replace the original model. Parenthesized ranges are 95% bootstrap intervals for the full-cohort mean-imputation sensitivity.

## Table S11C Frozen D2 scores evaluated against D1

| **Cohort** | **D1 n** | **Budget %** | **Tested n** | **Detected n** | **Recall %** | **Yield %** | **AUROC** | **AUPRC** |
| --- | --- | --- | --- | --- | --- | --- | --- | --- |
| Development | 362 | 10 | 117 | 91 | 25.1 | 77.8 | 0.778 | 0.630 |
| Development | 362 | 20 | 234 | 157 | 43.4 | 67.1 | 0.778 | 0.630 |
| Development | 362 | 30 | 351 | 212 | 58.6 | 60.4 | 0.778 | 0.630 |
| Development | 362 | 40 | 468 | 258 | 71.3 | 55.1 | 0.778 | 0.630 |
| Development | 362 | 50 | 585 | 290 | 80.1 | 49.6 | 0.778 | 0.630 |
| External | 259 | 10 | 161 | 61 | 23.6 | 37.9 | 0.731 | 0.356 |
| External | 259 | 20 | 323 | 113 | 43.6 | 35.0 | 0.731 | 0.356 |
| External | 259 | 30 | 485 | 149 | 57.5 | 30.7 | 0.731 | 0.356 |
| External | 259 | 40 | 647 | 173 | 66.8 | 26.7 | 0.731 | 0.356 |
| External | 259 | 50 | 809 | 201 | 77.6 | 24.8 | 0.731 | 0.356 |

D1=eGFRcys−eGFRcr≤−15 mL/min/1.73 m². This is exploratory recognition of a related phenotype, not a newly trained D1 model or independent disease confirmation. Scores remain those of the frozen D2 model. No multiplicity correction.

## Table S12A Repeat paired-measurement agreement

| **Cohort** | **Window** | **N** | **Agreement % (95% CI)** | **Kappa (95% CI)** |
| --- | --- | --- | --- | --- |
| External | 30-365 days | 53 | 86.8 (75.2, 93.5) | 0.457 (0.088, 0.779) |
| External | 7-365 days | 176 | 88.6 (83.1, 92.5) | 0.563 (0.382, 0.711) |
| Development | 30-365 days | 59 | 66.1 (53.4, 76.9) | 0.175 (-0.093, 0.436) |
| Development | 7-365 days | 74 | 68.9 (57.7, 78.3) | 0.246 (-0.004, 0.470) |

Primary window=30–365 days; 7–365 is sensitivity. Earliest valid same-specimen repeat, verified in original LIS. Agreement interval=Wilson; Kappa interval=2000 patient bootstrap. Follow-up is limited to the original extraction period; repeat availability is selective and does not validate true GFR accuracy.

## Table S12B Repeat D2 transitions and timing

| **Cohort** | **Window** | **Positive to positive** | **Positive to negative** | **Negative to positive** | **Negative to negative** | **Median days** | **Median change in eGFR difference** |
| --- | --- | --- | --- | --- | --- | --- | --- |
| Development | 30-365 days | 7 | 9 | 11 | 32 | 180.0 | -0.82 |
| Development | 7-365 days | 10 | 11 | 12 | 41 | 130.5 | -1.99 |
| External | 30-365 days | 4 | 4 | 3 | 42 | 61.0 | 2.99 |
| External | 7-365 days | 17 | 9 | 11 | 139 | 12.0 | 0.14 |

Transitions use index and earliest repeat D2. Difference change=(repeat eGFRcys−eGFRcr)−(index eGFRcys−eGFRcr), mL/min/1.73 m².

## Table S12C Repeat-subset selection at the 30–365 day window

| **Cohort** | **Variable** | **Repeat subset n** | **No qualifying repeat n** | **SMD** |
| --- | --- | --- | --- | --- |
| External | age | 53 | 1566 | 0.055 |
| External | sex | 53 | 1566 | 0.058 |
| External | eGFRcr_2021 | 53 | 1566 | 0.022 |
| External | D2 | 53 | 1566 | 0.274 |
| External | BUN | 53 | 1566 | -0.018 |
| External | ALB | 53 | 1566 | 0.040 |
| External | HGB | 53 | 1566 | -0.251 |
| External | PLT | 53 | 1566 | -0.091 |
| External | GLU | 53 | 1566 | 0.120 |
| Development | age | 59 | 1111 | 0.369 |
| Development | sex | 59 | 1111 | 0.079 |
| Development | eGFRcr_2021 | 59 | 1111 | -0.455 |
| Development | D2 | 59 | 1111 | 0.224 |
| Development | BUN | 59 | 1111 | 0.274 |
| Development | ALB | 59 | 1111 | -0.552 |
| Development | HGB | 59 | 1111 | -0.356 |
| Development | PLT | 59 | 1111 | -0.051 |
| Development | GLU | 59 | 1111 | -0.338 |

SMD=repeat subset minus no qualifying repeat in the final modeling cohort. Availability of repeat testing is selective, and observation is limited by the original extraction period. Sex is male proportion; D2 is the index phenotype. SMDs do not remove selection bias.

# Figure S1 Correlation structure of the pre-redundancy pool

Pairwise-complete Spearman correlations for 39 pre-redundancy variables in the development cohort (n = 1,170). No imputation was performed for this matrix. A/G used the uniform ALB/GLB definition. This is the stage before clinical redundancy review, not the final 28-variable pool. The correlation matrix was descriptive and did not automatically determine variable exclusion. Color limits are -1 to 1. WBC: white blood cells; RBC: red blood cells; HGB: hemoglobin; HCT: hematocrit; MCV: mean corpuscular volume; MCH: mean corpuscular hemoglobin; MCHC: mean corpuscular hemoglobin concentration; RDW: red-cell distribution width; PLT: platelets; MPV: mean platelet volume; NEUT/LYMPH/MONO/EO/BASO: neutrophils/lymphocytes/monocytes/eosinophils/basophils; #: absolute count; BUN: blood urea nitrogen; UA: uric acid; ALB: albumin; TP: total protein; GLB: globulin; A/G: albumin-to-globulin ratio; ALT/AST: alanine/aspartate aminotransferase; ALP: alkaline phosphatase; GGT: gamma-glutamyl transferase; TBIL: total bilirubin; GLU: glucose.

# Figure S2 Precision–recall comparison of seven Full models

Development pooled patient-level OOF precision-recall curves for seven algorithms using the identical 28-variable Full pool (n = 1,170; D2 = 214). AUPRC is average precision (AP), displayed in the legend. The dashed line represents cohort D2 prevalence. These are development/model-selection estimates from the same folds used for tuning and are not independent internal-validation estimates.

# Figure S3 Raw external XGBoost calibration

Original locked nine-variable XGBoost probabilities in the independent external validation cohort (n = 1,619; D2 = 112). Points show observed proportions against mean predictions in 10 quantile-based groups and are not connected. The green curve is the diagnostic logistic calibration fit using the original probabilities; the dashed diagonal represents ideal calibration. Both axes span 0-1. No recalibrated probability is used in this figure. Raw intercept = -2.209, slope = 0.848, CITL = -2.250, Brier = 0.144; mean prediction = 0.299 and observed prevalence = 0.069. These original-probability results remain distinct from the post-hoc cross-fitted local calibration assessment in Table S6 and Figure S4. Main Figure 3D displays original XGBoost and clinical-core calibration.

# Figure S4 Cross-fitted local recalibration sensitivity

Five-fold cross-fitted local logistic recalibration of original external XGBoost predictions in 1,619 participants (112 D2 events), retaining the historical stratified seed-42 folds. Parameters from four folds were applied to the held-out fold. This is external-cohort local-adaptation sensitivity, not independent external validation. Points show decile means and observed proportions; the solid line is the diagnostic logistic calibration fit and the diagonal is ideal calibration. Both axes span 0–1. The diagnostic curve outside observed prediction support is parametric extrapolation, not additional data. Brier=0.058.

# Figure S5 Exploratory DCA sensitivity

Original XGBoost, original clinical core and cross-fitted locally recalibrated XGBoost are shown with test-all and test-none in the same external cohort. Panel A shows the historical 0–12% display; panel B uses the fixed 0–50% descriptive range. Negative net benefits and reference curves remain visible. Neither range has an established project-specific clinical decision-threshold rationale. The local-adaptation result is a sensitivity analysis and is not a fair unadapted-model comparison. Pointwise intervals and their conditional uncertainty are in Table S6C.
